# Artificial Intelligence-Based Robust Hybrid Algorithm Design and Implementation for Real-Time Detection of Plant Diseases in Agricultural Environments

**DOI:** 10.3390/biology11121732

**Published:** 2022-11-29

**Authors:** İlayda Yağ, Aytaç Altan

**Affiliations:** Department of Electrical Electronics Engineering, Zonguldak Bülent Ecevit University, Zonguldak 67100, Turkey

**Keywords:** agricultural plant, plant leaf disease classification, 2D discrete wavelet transform, 2D signal processing, flower pollination optimization, artificial intelligence, real-time detection

## Abstract

**Simple Summary:**

Plant disease, defined as an abnormal condition that disrupts the normal growth of the plant, is one of the main causes of economic losses in the agricultural industry. Early diagnosis of plant disease is critical to increasing agricultural crop productivity. In this paper, a new robust hybrid classification model based on swarm optimization-supported feature selection, including machine learning and deep learning algorithms, that allows real-time classification of diseases in apple, grape, and tomato plants has been developed. In this way, it will be possible to diagnose the plant disease at an early phase and apply the appropriate treatment.

**Abstract:**

The early detection and prevention of plant diseases that are an important cause of famine and food insecurity worldwide are very important for increasing agricultural product productivity. Not only the early detection of the plant disease but also the determination of its type play a critical role in determining the appropriate treatment. The fact that visual inspection, which is frequently used in determining plant disease and types, is tiring and prone to human error, necessitated the development of algorithms that can automatically classify plant disease with high accuracy and low computational cost. In this study, a new hybrid plant leaf disease classification model with high accuracy and low computational complexity, consisting of the wrapper approach, including the flower pollination algorithm (FPA) and support vector machine (SVM), and a convolutional neural network (CNN) classifier, is developed with a wrapper-based feature selection approach using metaheuristic optimization techniques. The features of the image dataset consisting of apple, grape, and tomato plants have been extracted by a two-dimensional discrete wavelet transform (2D-DWT) using wavelet families such as biorthogonal, Coiflets, Daubechies, Fejer–Korovkin, and symlets. Features that keep classifier performance high for each family are selected by the wrapper approach, consisting of the population-based metaheuristics FPA and SVM. The performance of the proposed optimization algorithm is compared with the particle swarm optimization (PSO) algorithm. Afterwards, the classification performance is obtained by using the lowest number of features that can keep the classification performance high for the CNN classifier. The CNN classifier with a single layer of classification without a feature extraction layer is used to minimize the complexity of the model and to deal with the model hyperparameter problem. The obtained model is embedded in the NVIDIA Jetson Nano developer kit on the unmanned aerial vehicle (UAV), and real-time classification tests are performed on apple, grape, and tomato plants. The experimental results obtained show that the proposed model classifies the specified plant leaf diseases in real time with high accuracy. Moreover, it is concluded that the robust hybrid classification model, which is created by selecting the lowest number of features with the optimization algorithm with low computational complexity, can classify plant leaf diseases in real time with precision.

## 1. Introduction

Due to the rapid increase in the world population and the current population size, efficient production of agricultural products is needed, as well as the efficient use of agricultural lands, which are limited. In the efficient production of agricultural products, it is critical to detect plant diseases at an early stage, as well as spraying, fertilizing, and weed detection processes. It is known that losses in agricultural product yield due to plant diseases range between 20% and 40%. This heavy yield loss also means a reduced market volume for buyers of that product [1]. A delay in the diagnosis of viral, pathogenic, or plague-borne plant diseases requires the application of more doses of pesticides to the diseased plant, thus resulting in a decrease in crop quality [2]. Detecting and correctly classifying plant diseases at an early stage not only contributes to improving the quality of agricultural products but also allows for the reduction of undesirable chemical spray applications such as fungicides and herbicides [3]. In this study, we propose a robust hybrid plant disease classification model based on metaheuristic optimization-assisted feature selection that includes machine learning and deep learning algorithms for real-time early detection of diseases in apple, grape, and tomato plants. The performance of the model embedded in the NVIDIA Jetson Nano developer kit on an unmanned aerial vehicle (UAV) has been experimentally tested on apple, grape, and tomato plants.

Plant disease, which is one of the main causes of economic losses in the agricultural industry in the world, is defined as an abnormal state that disrupts the normal growth of the plant [4]. Symptoms related to the abnormal state can usually be seen in the leaf, stem, and root parts of the plant. Leaf images are a good source of information in the classification of plant diseases. Therefore, researchers focus on the classification of plant diseases through leaf images [5]. In this study, plant diseases are classified using leaf images of apple, grape, and tomato plants.

Although traditional methods based on visual inspection are used in the detection of plant diseases in small-scale agricultural lands, it is very difficult to apply these methods in large-scale agricultural lands as they require tiring and continuous monitoring [6]. Especially in diagnosing both the species and the disease of plants with similar leaves, this process becomes more difficult and leads to visual errors. To cope with these challenges, various image processing and artificial intelligence techniques such as machine learning and deep learning are used in the real-time classification of plant diseases based on plant leaf images [7,8,9].

In the literature, there are image processing-based studies for real-time prediction of plant diseases and disease levels on many plant species. In [3], the severity level of white-tip disease (*Aphelenchoides besseyi Christie*), commonly seen in paddy crops, is estimated based on image processing techniques. The diseased area in the paddy crop is determined with the help of a color image segmentation algorithm based on chromatic aberration. The severity of the disease is estimated by proportioning the area of the determined region to the entire area of the leaf. Based on the estimation results obtained, a variable rate chemical spray system has been developed for the precise application of agrochemicals in real time. In [10], a ΔE (Delta E) segmentation-based feature extraction algorithm is proposed to classify diseases in images of citrus plants. The diseased region is identified by the ΔE method and hue saturation value (HSV), local binary patterns (LBP), and red green blue (RGB) histogram models are extracted from this region. The features obtained with the HSV, LBP, and RGB descriptors are combined, and a hybrid feature set is created. Principle component analysis (PCA) is used to reduce dimensionality. Fine k-nearest neighbor (kNN), cubic support vector machine (SVM), boosted tree, and bagged tree ensemble classifiers are tested. It is stated that the best performance is achieved with the bagged tree ensemble classifier. In [11], the temperature difference between the healthy and infected parts of the cucumber leaf is measured and downy mildew disease is detected using Fourier transform infrared (FTIR) spectroscopy. It is reported that the FTIR technique is effective in the pre-symptomatic detection of downy mildew disease on cucumber leaves. In [12], feature extraction and segmentation algorithms are used together for detection and classification of early scorch disease in banana leaves, fungal disease in bean leaves, sunburn disease in lemon leaves, and bacterial disease in roses. Segmentation of diseased regions has been carried out using a genetic algorithm (GA). It is also emphasized that the optimization algorithm used reduces the computational complexity.

In the image processing approach, the classification accuracy of plant diseases directly depends on the performance of feature extraction and segmentation. Especially, image segmentation plays a key role in detecting the severity of leaf disease, and the segmentation precision of leaf disease determines the accuracy of the classification of disease severity [13]. Although image processing techniques give good results in determining the severity of a disease in plant leaves, they cannot show the same success in classifying disease types in plant leaves where there is more than one disease type. Instead, artificial intelligence-based approaches, which use features with higher recognition accuracy through iterative learning without the need for specific features extracted by image processing techniques, have recently been frequently used in the classification of plant leaf diseases [14]. In [15], multiple types of tomato leaf diseases are classified using both deep learning and machine learning algorithms. To apply the machine learning models, a total of 52 texture features are extracted using a gray level co-occurrence matrix (GLCM) and local binary pattern (LBP) techniques, and 105 color features are extracted using color histograms and color moment approaches. The performances of kNN, SVM, and random forest (RF) from machine learning algorithms and AlexNet, VGG16, ResNet34, EfficientNet-b0, and MobileNetV2 architectures from deep learning algorithms are tested for 10 different types of tomato leaf disease. In [8], a set of images containing 12 plant species is separated into training and testing parts, and a convolutional neural network (CNN) is trained with the help of training data for the classification of plant diseases. It has been reported that the accuracy performance of the obtained model varies between 60% and 100% depending on the disease characteristics of each plant species. In [16], a deep learning model based on the pruned version of MobileNet architecture is proposed for efficient classification of multiple plant diseases based on leaf images of healthy and diseased plants. The proposed model is compared with VGG and classical MobileNet architectures. It is stated that the proposed architecture achieves 98.34% classification accuracy with six times fewer parameters compared to MobileNet and 29 times fewer than VGG. The performance of different optimizers is tested to reduce the computational overhead of the model. It is indicated that Adam and Nadam optimizers’ convergence rates are faster than stochastic gradient descent (SGD) and contribute to the reduction of computational cost. Besides these studies, there are many studies on the recognition and classification of leaf diseases of many plant species with artificial intelligence algorithms such as machine learning and deep learning techniques [17,18,19,20].

Although the classification accuracy of the techniques mentioned in the classification of plant leaf diseases belonging to more than one species is high, the high model complexity and high computational costs make their use difficult in real-time applications. In the real-time classification problem of plant leaf diseases, optimal values of the hyperparameters of artificial intelligence-based models built with specific architectures are determined using swarm-based optimization algorithms to overcome these challenges [21]. CNN models, which have been frequently used in the automatic diagnosis and classification of plant leaf diseases in recent years, try to solve the hyperparameter problem with various metaheuristic optimization algorithms. Although this contributes to reducing the parameter complexity of the model, it increases the computational load of the model.

This study is robust and can classify plant leaf diseases in real time with high accuracy, a low parameter complexity, and a low computational cost, using the feature-based robust hybrid classification technique, including the wrapper approach consisting of the flower pollination algorithm (FPA), SVM, and a CNN classifier. The study considers feature extraction with signal processing techniques as a preprocessing step, so that the computational load of the model is not increased. Until now, features have been extracted with many signal processing techniques in the classification of plant leaf diseases, but feature extraction has not been performed with the two-dimensional discrete wavelet transform (2D-DWT) signal processing techniques based on various wavelet families. The distinguishing features defining the characteristic features of plant leaf diseases are extracted for the first time in this study with the 2D-DWT technique using wavelet families, such as biorthogonal, Coiflets, Daubechies, Fejer–Korovkin, and symlets. The main contributions of this research can be summarized as follows:

- A robust hybrid model based on 2D-DWT is proposed for the real-time classification of plant leaf diseases with high accuracy.
- Feature groups are extracted for each family by applying 2D-DWT with the biorthogonal, Coiflet, Daubechies, Fejer–Korovkin, and symlet wavelet families to the image dataset consisting of apple, grape, and tomato plants. The extracted feature groups for each wavelet family consist of distinctive features representing each plant leaf disease.
- The features that keep classifier performance high for each wavelet family are selected by the wrapper approach, consisting of the population-based metaheuristic FPA and SVM algorithms. The fitness function is computed by considering both the number of features used in the model and the model’s performance in order to keep the model’s complexity and computation cost at a minimum level.
- The efficiency of the proposed optimization algorithm is determined by comparing it with the particle swarm optimization (PSO) algorithm.
- To overcome the model hyperparameter problem, the CNN classifier is used, which only has a classification layer without a feature extraction layer and uses the lowest number of features that can keep classification performance high.
- For the real-time plant leaf disease classification problem, the model with the best performance is proposed, which includes the 2D-DWT signal processing method based on the “***sym7***” wavelet family, the wrapper approach consisting of FPA and SVM, and a CNN classifier.
- The proposed model is embedded in the NVIDIA Jetson Nano developer kit on the UAV. Real-time classification tests have been performed on apple, grape, and tomato plants to demonstrate that the proposed model can classify plant leaf diseases in real time with high accuracy.
- The experimental results obtained show that the model has low computational complexity and a minimum computational load; therefore, it can be used in real-time applications that require high classification accuracy.

The rest of this paper is structured as follows. The entire methodology that makes up the plant leaf disease classification model, including 2D-DWT, wavelet families, the wrapper approach consisting of the population-based metaheuristic FPA and SVM algorithms, CNN classifier, and classification performance metrics, is introduced in Section 2. The experimental results of the study, along with their detailed discussion, are presented in Section 3. Finally, Section 4 summarizes the concluding remarks and points to future work for research.

## 2. Framework of the Plant Diseases Detection Algorithm

This section describes our model structure that can classify leaf diseases of apple, grape, and tomato plants in real time with high accuracy. In our proposed model, features are extracted for each family by applying 2D-DWT signal processing techniques with the biorthogonal, Coiflet, Daubechies, Fejer–Korovkin, and symlet wavelet families to the image dataset consisting of apple, grape, and tomato plants. The features that provide the highest model performance and the lowest model complexity are selected with the wrapper approach, consisting of the FPA and SVM algorithms. The CNN classifier, which is used to overcome the model hyperparameter problem, classifies plant leaf diseases with the help of selected features. All the methodology used in our model is described in detail in the following subsections.

### 2.1. Discrete Wavelet Transform

The Fourier transform (FT), which has been successfully applied for stationary signals, cannot be used for non-stationary signals. Since the spectrum changes with time, FT is insufficient to reveal the correct spectrum for non-stationary signals. Non-stationary signals are divided into sufficiently small pieces by short-term Fourier transform (STFT), and these small pieces are considered stationary. A time-frequency representation is needed to know what frequency components are present at different times and how they change as time passes. The sinusoidal frequency and phase content of a time-varying signal are found by STFT, and the relationship between frequency and time variation is defined with the help of a moving window. However, this technique is quite complicated to study non-stationary signals. Wavelet transform (WT) offers an alternative approach to overcome such challenges of FT and STFT with its variable-size windowing techniques [22,23]. The frequency and time information about a signal is obtained with this approach when short time intervals at high frequencies and long-time intervals at low frequencies can be selected. The wavelet transform is a transform technique that separates signals into different frequency components and examines each component with its resolution at that scale. Wavelets provide a good tool for time-frequency analysis [24].

**Definition** **1.** *If* ψ(t) *is a real-valued function whose Fourier spectrum,* ψ(s) *satisfies the admissibility criterion.*

(1) $$C_{\psi }=\int_{-\infty }^{+\infty }\frac{{\left|\psi \left(s\right)\right|}^{2}}{\left|s\right|}ds<\infty$$

*then, *ψ(t)* is called a basic wavelet. Notice that, due to the*s* in the denominator of the integrand, it is necessary that:*

(2) $$\psi \left(0\right)=0\Rightarrow \int_{-\infty }^{+\infty }\psi \left(t\right)dt=0$$

*Furthermore, since* ψ(∞)=0 *as well, we can see that the amplitude spectrum of an admissible wavelet is similar to the transfer function of a bandpass filter.*

*A set of wavelet basis functions, *$\left\{\psi_{a,b}\left(t\right)\right\}$*, can be generated by translating and scaling the basic wavelet, *ψ(t)*, as:*

(3) $$\psi_{a,b}\left(t\right)=\frac{1}{\sqrt{a}}\;\psi \left(\frac{t-b}{a}\right)$$

*where* a>0 *and* b *are real numbers. The variable* a *reflects the scale of a particular basis function, while* b *specifies its translated position along the* t.

*The continuous wavelet transform (CWT) of* x(t) *with respect to the wavelet* ψ(t) *is then:*

(4) $$W\left(a,b\right)=\langle x,\psi_{a,b}\rangle =\int_{-\infty }^{+\infty }x\left(t\right)\psi_{a,b}^{*}\left(t\right)dt$$

*The wavelet transform coefficients are given as inner products of the function being transformed with each of the basis functions.*

*The inverse continuous wavelet transform is mathematically defined as* [25]*:*

(5) $$f\left(t\right)=\frac{1}{C_{\psi }}=\int_{0}^{+\infty }\int_{-\infty }^{+\infty }W\left(a,b\right)\psi_{a,b}\left(t\right)db\frac{da}{a^{2}}$$

*where* $C_{\psi }$ *is a constant of the wavelet used.*

*When we take the scaling and shifting wavelet parameters as dyadic variables* m *and* n *in discrete time, the* *discrete wavelet transform (DWT) of the signal is defined as:*

(6) $$W\left(m,n\right)=\langle x,\psi_{m,n}\rangle =a_{0}^{-m/2}\underset{k}{\sum }f\left[k\right]\psi^{*}\left(\frac{k-na_{0}^{m}b_{0}}{a_{0}^{m}}\right)$$

*where* m *and* n *denote frequency and time localizations, respectively. Here,* $a_{0}>1$ *,* $b_{0}>0$ *and* m, n ∈ Z *, the scaling and shifting parameters are represented as* $a_{0}^{m}$ *,* $na_{0}^{m}b_{0}$ *, respectively.*

### 2.2. Multiresolution Analysis

Not only the time domain features of the signals, but also the frequency domain features can be extracted and classified. DWT-based multi-resolution analysis is very useful in feature extraction applications from image signals. Undesirable components, such as noise and trend in the signal, can be separated by multiresolution analysis (MRA) [26].

**Definition** **2.** *Analysis of signals in terms of both time and frequency is performed by computing scaling and wavelet functions. The scaling* $\left\{\varphi_{m,n}\left[k\right]\right\}$ *and wavelet* $\left\{\psi_{m,n}\left[k\right]\right\}$ *functions are given by:*

(7) $$\varphi_{m,n}\left[k\right]=2^{-m/2}\varphi \left(2^{-m}k-n\right)$$

(8) $$\psi_{m,n}\left[k\right]=2^{-m/2}\psi \left(2^{-m}k-n\right)$$

*where* m, n ∈ Z *. The high-scale, low-frequency components correspond to approximation coefficient* {φ[k]} *that bridge the wavelets and filter banks and is denoted by:*

(9) $$\varphi \left[k\right]=\underset{k}{\sum }f\left[k\right]h\left[k-2n\right]$$

*where* h[n] *corresponds to the low pass filter that is associated with the scaling function. The low-scale high-frequency components correspond to detail coefficient* {ψ[k]} *is expressed by:*

(10) $$\psi \left[k\right]=\underset{k}{\sum }f\left[k\right]g\left[k-2n\right]$$

*where* g[n] *is the complementary high pass filter in this orthogonal filter bank. The scaling function, defined by the filter coefficients* h[n] *, provides approximation coefficients* {φ[k]} *, which are also referred to as low-pass output. The wavelet function, defined by the filter coefficients* g[n] *, provides the detailed coefficients* {ψ[k]} *, or alternatively the high-pass output.*

### 2.3. Wavelet Families

The biorthogonal, Coiflet, Daubechies, Fejer–Korovkin, and symlet wavelet families used in the calculation of high and low pass filter coefficients of the DWT are introduced in the following subsections.

#### 2.3.1. Biorthogonal Wavelet

Biorthogonal wavelets that are not based on vanishing moments have a compactly supported symmetrical structure. In the biorthogonal case, rather than having one scaling and wavelet function, there are two scaling functions $\left\{\varphi ,\;\tilde{\varphi }\right\}$ that may generate different MRA, and accordingly two different wavelet functions $\left\{\psi ,\;\tilde{\psi }\right\}$. For orthogonal wavelets, recursively computations of the scaling function and mother wavelet are presented by [27]

(11) $$\varphi \left(t\right)=2\underset{k}{\sum }h_{0}\left(k\right)\varphi \left(2t-k\right)$$

(12) $$\tilde{\varphi }\left(t\right)=2\underset{k}{\sum }{\tilde{h}}_{0}\left(k\right)\tilde{\varphi }\left(2t-k\right)$$

(13) $$\psi \left(t\right)=2\underset{k}{\sum }g_{1}\left(k\right)\varphi \left(2t-k\right)$$

(14) $$\tilde{\psi }\left(t\right)=2\underset{k}{\sum }{\tilde{g}}_{1}\left(k\right)\tilde{\varphi }\left(2t-k\right)$$

where $h_{0}\left(k\right)$, ${\tilde{h}}_{0}\left(k\right)$, $g_{1}\left(k\right)$, and ${\tilde{g}}_{1}\left(k\right)$ are dual filter coefficients.

#### 2.3.2. Coiflet Wavelet

The Coiflet wavelet, designed by Ingrid Daubechies at the suggestion of Ronald Coifman, are discrete wavelets with vanishing moments and scaling functions. The idea behind Coiflet wavelets is to specify moment conditions approaching zero in the associated scaling function. This situation also causes the creation of a wavelet filter with descriptive properties [28]. The Coiflet wavelet function has 2N moments equal to zero, and the scaling function has 2N−1 moments equal to zero. The two functions have a support of the length of 6N−1. It is more symmetrical than compared to Daubechies wavelets. The general characteristic of the Coiflet wavelet is that for any given support width, it has the highest number of vanishing moments for both the scaling {φ} and the wavelet function {ψ}. The lengths of the scaling and wavelet functions for Coiflet wavelets are calculated as (L = 6, 12, 18, 24, 30). The approximation features also depend on the number of vanishing wavelet moments [29].

It is sufficient to define the Coiflets in terms of the H filter. Since for any integer n the filter $z^{n}H\left(z\right)$ generates the same MRA as H, we always assume the coefficients $\left\{h_{k}\right\}$ of H to be zero for k<0.

**Definition** **3.** *Let* ${\left\{h_{j}\right\}}_{j=0}^{L-1}$ *be the coefficients of a real quadrature mirror filter* H *. We say that* H *is a Coiflet of shift* γ *and moments* M, N *if the conditions in Equations (15)–(17) are satisfied:*

(15) $$\overset{L-1}{\underset{j=0}{\sum }}{\left(-1\right)}^{j}j^{k}h_{j}=0\;\;\;\;\;for\;\;0\leq k<M$$

(16) $$\overset{L-1}{\underset{j=0}{\sum }}j^{k}h_{j}=\gamma^{k}\;\;\;\;\;\;\;\;\;\;\;\;for\;\;0\leq k<N$$

(17) 3M>L−1             and   3N≥L−1

*If a Coiflet* H *also satisfies Cohen’s condition that* H *is non-zero in certain locations on the unit circle* [30]*, its associated wavelet and scaling functions will have vanishing moments* M *and* N−1*, respectively* [28]*. The normalization* H(1)=1 *corresponds to Equation (16) with* k=0.

#### 2.3.3. Daubechies Wavelet

Daubechies wavelets represent a wavelet summation that improves the frequency domain properties of Haar wavelets. These wavelets are a family of orthogonal wavelets indexed with N to represent the number of N vanishing wavelet moments. The Daubechies wavelets have two important properties. First, it has a finite number of non-zero $p_{m}$ scaling coefficients. This means that the scaling and wavelet functions are compact. The N-order Daubechies scaling function has 2N nonzero scaling coefficients, and the support size width of the scaling and wavelet functions is in the range [0, 2N−1 ]. The second property of the N-order Daubechies wavelet is that the initial N−1 moments of the wavelets are zero [31].

The moments of the scaling and wavelet functions are defined by:

(18) $$\int_{-\infty }^{+\infty }\varphi \left(k\right)k^{j}d\left(k\right)$$

and:

(19) $$\int_{-\infty }^{+\infty }\psi \left(k\right)k^{j}d\left(k\right)$$

respectively, where φ(k) is the scaling function and ψ(k) is the wavelet function. The moment of the Daubechies wavelets is expressed as:

(20) $$\int_{-\infty }^{+\infty }\psi_{N}\left(k\right)k^{j}d\left(k\right)=0\;\;\;\;\;for\;j=0,\ldots ,\;N-1$$

The zeroing the first N moment of the $\psi_{N}$ wavelet is often abbreviated as $\psi_{N}$, and has N zeroing moments. The zeroed moments mean that every N-order Daubechies wavelet basis function is orthogonal to all polynomials of degrees less than N. Daubechies wavelets are classified according to the number of zero moments they have. The smoothness of the scaling and wavelet functions increases in direct proportion to the number of zero moments [32].

(21) $$\varphi \left(k\right)=\sqrt{2}\overset{N-1}{\underset{m=0}{\sum }}p_{m}\varphi \left(2k-m\right)$$

The wavelets ψ(k) are defined in terms of the scaling function. The expression relating the wavelet to the scaling function is:

(22) $$\psi \left(k\right)=\overset{N-1}{\underset{m=0}{\sum }}{\left(-1\right)}^{m}r_{N-1-m}\varphi \left(2k-m\right)$$

where N represents the order of the system, m denotes the localization parameter, $p_{m}$ and $r_{N-1-m}$ are the vector of scaling and wavelet filter coefficients, respectively.

#### 2.3.4. Fejer–Korovkin Wavelet

The kernel function of the Fejer–Korovkin wavelet, which is more symmetrical but less smooth than the Daubechies filters, is calculated with the help of Equations (23)–(25). A special class of filters is the family of filters associated with MRA filters. An MRA filter $m_{0}$ generates the scaling function, associated with the MRA by [33]:

(23) $$\hat{\varphi }\left(\xi \right)=\overset{\infty }{\underset{j=1}{\prod }}m_{0}\left(2^{-j}\xi \right)$$

A sufficient condition for a filter $m_{0}$ satisfying Equation (24), and taking on the value 1 at 0, to be an MRA filter is that $m_{0}$ does not vanish on [−π/2, π/2].

(24) $${\left|m_{0}\left(\xi \right)\right|}^{2}+{\left|m_{0}\left(\xi +\pi \right)\right|}^{2}=1$$

The kernel function {K(ξ)} is expressed by:

(25) $$K\left(\xi \right)=1+\pi \overset{N-1}{\underset{l=0}{\sum }}{\left(-1\right)}^{l}\left(2l+1\right)a_{1}cos\left(\left(2l+1\right)\xi \right)$$

where $a_{1}$ is sequence of coefficients and ξ is index of vector. The Fejer–Korovkin filters are defined by:

(26) $${\left|m_{0}^{n}\left(\xi \right)\right|}^{2}=\frac{1}{2\pi }\int_{-\pi /2}^{\pi /2}K\left(\xi -u\right)du$$

where $m_{0}^{n}$ has length n if n is even and length n+1 if n is odd.

#### 2.3.5. Symlets Wavelet

Symlets wavelets, which are similar to the structure of Daubechies wavelets, are called orthogonal, biorthogonal, and least asymmetrical wavelets, and were introduced to the literature by Daubechies [29]. Daubechies wavelets have a maximum phase, while Symlets wavelets have a minimum phase. Unlike Daubechies wavelets, Symlets wavelets have a smoothed wavelet function with near-zero moments [34]. Symlets are more symmetrical than Daubechies, even though they have a 2N−1 support size with N vanishing moments. Symlet wavelet coefficients for different filter lengths are calculated in [29].

### 2.4. Two-Dimensional Discrete Wavelet Transform (2D-DWT)

To simplify the vortex dynamics within a given implementation of the vorticity field, 2D-DWT is used, assuming that the vorticity field has finite energy; i.e., $\int \zeta^{2}\left(x\right)dx<\infty$. Let φ be a scaling function and ψ the corresponding wavelet forming an orthonormal basis on $L^{2}\left(R\right)$.

Three separable, “directionally sensitive” 2D-wavelet functions $\psi^{H}\left(x,y\right)$, $\psi^{V}\left(x,y\right)$, and $\psi^{D}\left(x,y\right)$ can be defined as

(27) $$\psi^{H}\left(x,y\right)=\psi \left(x\right)\varphi \left(y\right)$$

(28) $$\psi^{V}\left(x,y\right)=\varphi \left(x\right)\psi \left(y\right)$$

(29) $$\psi^{D}\left(x,y\right)=\psi \left(x\right)\psi \left(y\right)$$

corresponding to horizontal, vertical, and diagonal directions, respectively. The directional sensitivity is a natural consequence of the separability in Equations (27)–(29); it does not increase the computational complexity of the 2D-DWT with the biorthogonal, Coiflet, Daubechies, Fejer–Korovkin, and symlet wavelet families used in this study.

The separable scaling function φ(x,y)=φ(x)φ(y) is associated with the approximation space. The separable 2D-scaling and frequency dependent wavelet functions are defined as [35]:

(30) $$\varphi_{j,m,n}\left(x,y\right)=2^{j/2}\varphi \left(2^{j}x-m,\;2^{j}y-n\right)$$

(31) $$\psi_{j,m,n}^{i}\left(x,y\right)=2^{j/2}\psi \left(2^{j}x-m,\;2^{j}y-n\right)$$

where i term denotes one of H, V, or D. The 2D-DWT calculation for the function f(x,y) in M and N dimensions is expressed by:

(32) $$W_{\varphi }\left(j_{0},m,n\right)=\frac{1}{\sqrt{MN}}\overset{M-1}{\underset{x=0}{\sum }}\overset{N-1}{\underset{y=0}{\sum }}f\left(x,y\right)\varphi_{j_{0},m,n}\left(x,y\right)$$

(33) $$W_{\psi }^{i}\left(j,m,n\right)=\frac{1}{\sqrt{MN}}\overset{M-1}{\underset{x=0}{\sum }}\overset{N-1}{\underset{y=0}{\sum }}f\left(x,y\right)\psi_{j_{0},m,n}^{i}\left(x,y\right)$$

for j=0,1, …,J−1 and $m,n=0,1,2,\;\ldots ,\;2^{j-1}$. Note that $j_{0}$ is an arbitrary starting scale. The wavelet transform coefficients defined by Equations (32) and (33) are called approximation and detail coefficients, respectively. $W_{\varphi }\left(j_{0},m,n\right)$ coefficients describe an approximation of f(x,y) at this scale, and $W_{\psi }^{i}\left(j,m,n\right)$ coefficients compose diagonal, vertical, and horizontal details for scales $j\geq j_{0}$.

In this study, 2D-DWT with the biorthogonal, Coiflet, Daubechies, Fejer–Korovkin, and symlet wavelet families is applied to the image dataset consisting of apple, grape, and tomato plants. The feature groups extracted at various filter lengths for each wavelet family are used in the proposed plant leaf disease classification model structure. It should be noted that all of the wavelets used in the work avoid the computational complexity of complex numbers.

The scaling function, horizontal, vertical, and diagonal wavelet functions of wavelets that provide the best classification performance for each wavelet family are presented in Table 1. In the study, the wavelets that provide the best classification performance are obtained as “***bior2.4***” for the biorthogonal spline wavelet family, “***coif1***” for the Coiflet wavelet family, “***db5***” for the Daubechies wavelet family, “***fk18***” for the Fejer–Korovkin wavelet family, and “***sym7***” for the symlet wavelet family. Moreover, the best classification performance is obtained by the features extracted with “***sym7***” in the study, where there are twelve classes consisting of one healthy and three disease classes for each of the three plant species consisting of apple, grape, and tomato.

### 2.5. Flower Pollination Algorithm

**Figure 1:**
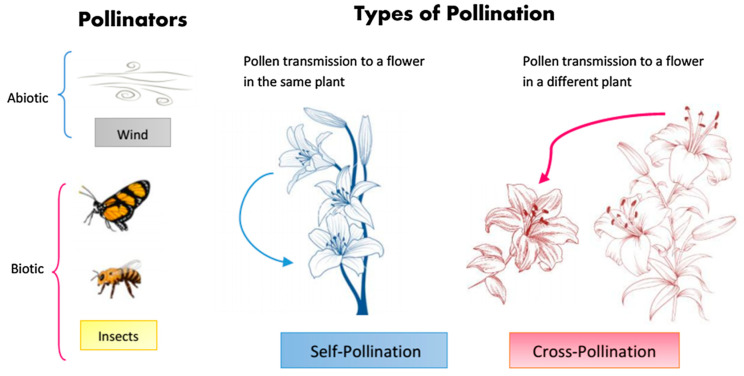
Pollination types and pollinators in the FPA [38].

The FPA introduced by Xin-She Yang in 2012 is a new metaheuristic inspired by the reproductive process of flowering plants to achieve the best result in the shortest time in solving global optimization problems [36]. Briefly, the algorithm mimics pollination in plants, including all the mechanisms for pollinators such as pollen transfer insects, bats, birds, wind, and water. The main purpose of flower pollination is to provide optimal vitality and the optimum biological reproduction phase. Pollination and other factors interact best to reproduce plants. Flowers need pollinators to carry out their pollination guidelines. There are two types of pollinators. The first of these are biotic pollinators, such as bats, flies, and bees. These pollinators can carry the pollen of flowers over great distances. In addition, the flight paths of these pollinators can be modeled with the Lévy distribution. The second type of pollinator is abiotic pollinators, such as water and wind. This form of pollination is considered to occur over short distances [37]. The pollination process is modeled by dividing it into two groups as cross-pollination and self-pollination. Cross-pollination is considered as the flowers of different plants pollinate each other. Self-pollination is defined as the pollination of the same flowers or different flowers of a plant. Self-pollination is used as the local search directive, while biotic cross pollination is defined as the global search directive. In the self-pollination process, there are some factors, such as the proximity of flowers to each other and the wind. Because of these factors, the local search process has more weight in the general search process. The pollinators and pollination types used in FPA are shown in Figure 1. The pollination process is mimicked using the following four basic rules:

(i) Global pollination processes are carried out biotically, and pollinators carry pollen in the form of cross-pollination according to their Lévy flight.
(ii) Abiotic pollination can occur in abiotic conditions, such as self-pollination and wind diffusion as local pollination.
(iii) The coefficient called flower constancy is expressed as the probability of reproduction and varies in proportion to the similarity of flower species.
(iv) Global pollination and local pollination are controlled by a switch probability p ∈ [0, 1]. It is noted that p indicates the percentage balance between local and global search in the optimization search field.

In global pollination, pollen can be carried long distances because insects can fly for a long time. This guarantees the best reproduction. The mathematical expression of global pollination and flower constancy is defined by:

(34) $$X_{i}^{t+1}=X_{i}^{t}+L\left(X_{i}^{t}-g_{*}\right)$$

where $X_{i}^{t}$ is the solution vector at iteration t, $g_{*}$ is the current best solution among all solutions in the current iteration, i is the pollen bundle or solution vector index, L is the step size representing the strength of pollination.

As insects and birds fly for a long distance, their motion can be shown according to the Lévy distribution. The long distances taken by pollinators are imitated by Lévy flight. Lévy flight distribution is defined as:

(35) $$L\sim \frac{\lambda \Gamma \left(\lambda \right)\sin\left(\frac{\pi \lambda }{2}\right)}{\pi }\frac{1}{s^{1+\lambda }}\begin{array}{cc} & \left(s\gg s_{0}>0\right) \end{array}$$

where Γ(λ) is the standard gamma function with an index λ and Lévy distribution is valid for large steps s>0. The abiotic pollination and flower constancy in the algorithm can be represented as:

(36) $$X_{i}^{t+1}=X_{i}^{t}+\varepsilon \left(X_{j}^{t}-X_{k}^{t}\right)$$

where $X_{j}^{t}$ and $X_{k}^{t}$ represent pollen bundles of different flowers of the same plant; that is, different solutions of the solution set, ε denotes a random local pollination distance and has a normal distribution between [0, 1].

The remarkable feature of this algorithm is that many solution points are searched in the search space using the Lévy distribution. Determining the solution points in the solution space with the global search and searching the neighborhood of the solution points through the local search constitute the optimization logic of the algorithm.

The computational complexity of the wrapper approach, which consists of the FPA and SVM algorithms proposed from the study, is O(N(n×p+fn×p)). Here, N denotes the number of iterations, n indicates the number of features, and fn defines fitness function value, p represents the number of selected features. Since FPA has fast convergence, low complexity, and good optimization performance, SVM has low computational complexity and is easily repeatable, the wrapper approach consisting of the FPA and SVM algorithms is used for feature selection in this study.

### 2.6. Convolutional Neural Network Classifier

Traditional neural networks are heavily inspired by the way biological neural systems work and consist of a high number of interconnected computational nodes working in a distributed manner to collectively learn from the input to optimize the final output. CNNs are similar to traditional neural networks in that they consist of neurons that self-optimize through learning. CNNs, which are used in many computer-vision fields, especially classification, include convolutional feature extraction and classification layers, unlike traditional neural networks [39]. In this study, the CNN classifier, which has only one classification layer without a feature extraction layer, is used to overcome the model hyperparameter problem and minimize the model complexity.

CNNs consist of three types of layers, the convolution layer, the pool layer, and the fully connected layer. The feature maps created in the final convolution and pooling layers are converted to a one-dimensional array of numbers and mapped to the final output of the network by connecting to one or more fully connected layers. In this study, the CNN classifier with two hidden layers consisting of flattened and fully connected layers is used. The features selected by the wrapper approach consisting of the FPA and SVM algorithms are given as input to the flatten layer of the CNN. Thus, the computational load of the model is prevented from increasing.

### 2.7. Performance Metrics for Classification

The whole dataset used in the study is randomly separated into two independent datasets, 80% and 20%, for the training and validation phases, respectively. The training performance of the models created in the study for plant leaf disease classification is measured by the *10-fold cross-validation* method. The training performance of each classification model is calculated by averaging the accuracy values for each fold. The performances of all classification models used in the study are measured on the test data with the help of the metrics in Equations (37)–(40). The metrics given in Equations (37)–(40) are calculated based on the confusion matrix. Here, TP, FP, TN, and FN denote true positives, false positives, true negatives, and false negatives, respectively.

(37) $$Accuracy=\frac{TP+TN}{TP+TN+FP+FN}{\;}^{,}\;$$

(38) $$Precision=\frac{TP}{TP+FP}{\;}^{,}$$

(39) $$Recall=\frac{TP}{TP+FN}{\;}^{,}$$

(40) $$F1\;score=\frac{2\times Recall\times Precision}{Recall+Precision}{\;}^{.}$$

Accuracy is generally a measure of how often the classifier estimates correctly. Precision is the ratio of how many positively predicted samples are predicted correctly. Recall is an indication of how many of the samples that should be predicted positively were predicted correctly. F1 score is the harmonic mean of recall and precision. It is a measure of how well the classifier is performing and is often used to compare classifiers [40].

### 2.8. Framework of the Proposed Methodology

**Figure 2:**
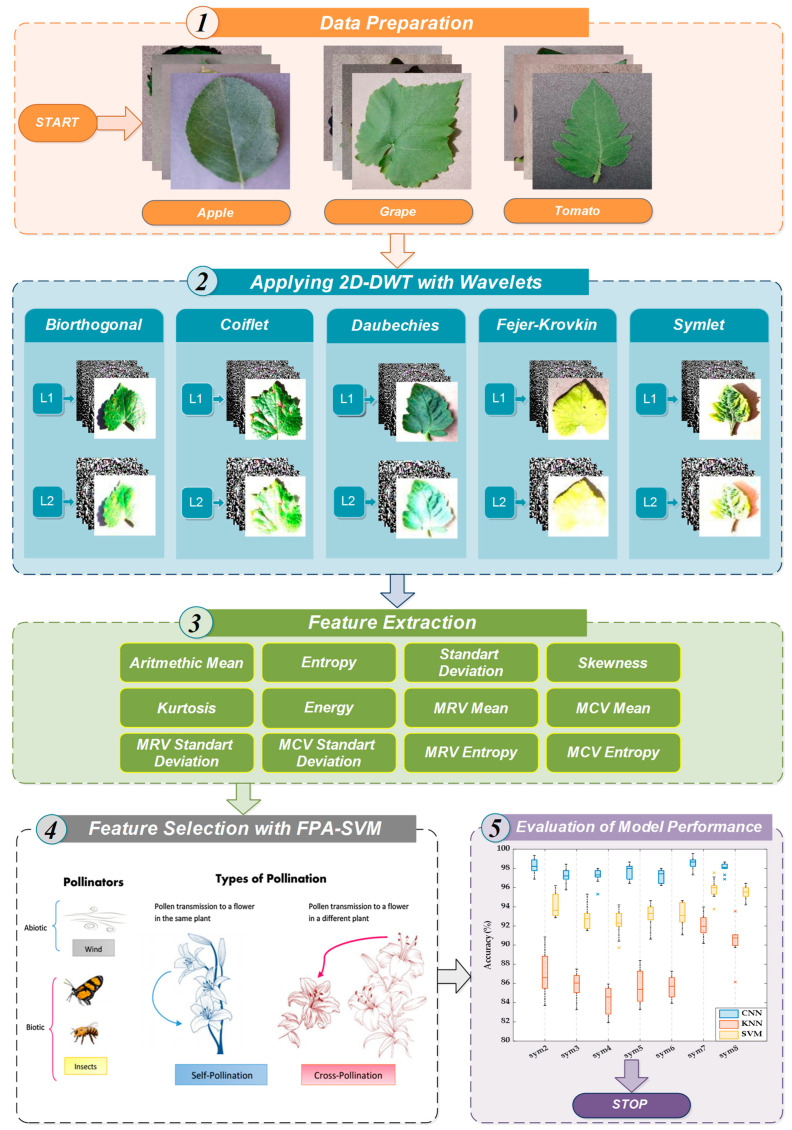
Framework of the proposed robust hybrid model to classify plant leaf diseases in real time.

In this study, a new robust hybrid model whose features are extracted with 2D-DWT and selected with the FPA-SVM wrapper approach and include a CNN classifier is proposed to classify plant leaf diseases in real time with high accuracy, low computational cost, and low parameter complexity. The framework of the proposed approach for real-time classification of plant leaf diseases is presented in Figure 2. The phases of the proposed approach are briefly summarized below:

***In the data preparation phase,*** the image dataset consisting of apple, grape, and tomato plant diseases is randomly divided into two independent datasets, 80% and 20%, respectively, for the training and validation phases.

***At the phase of applying 2D-DWT with wavelets,*** the distinguishing features defining the characteristic features of plant leaf diseases are extracted with the 2D-DWT using wavelet families such as biorthogonal, Coiflets, Daubechies, Fejer–Korovkin, and symlets.

***In the feature extraction phase,*** energy and statistical-based features are extracted from the vertical, horizontal, diagonal, and approximate matrices of 2D-DWT. Six features, namely the arithmetic mean, entropy, standard deviation, skewness, kurtosis, and energy, are applied to these four matrices. Moreover, the column vector, which is the maximum value of the columns of any matrices, is expressed with MCV, and the row vector, which is the maximum value of the rows of them, is expressed with MRV. Additionally, six properties, namely arithmetic mean, standard deviation, and entropy of both MCV and MRV, were applied to the four matrices. The same process is also repeated for the second level of decomposition in 2D-DWT. At the end of this phase, a total of 96 features are extracted.

***At the feature selection with the FPA-SVM phase,*** the most suitable ones among the normalized features for each wavelet family are selected with the help of the wrapper approach consisting of the FPA and SVM algorithms. In addition, the fitness function, which takes into account both the number of features used in the model and the model performance, is determined in order to keep the model complexity and computation cost to a minimum level.

***In the evaluation of model performance phase,*** CNN, SVM, and KNN classifier performances are measured with the help of performance metrics and the model with the highest performance is determined.

## 3. Experimental Results and Discussion

In this section, studies showing the classification efficiency of the proposed hybrid model for real-time classification of plant leaf diseases on a dataset consisting of leaf images of healthy and diseased apple, grape, and tomato plants are presented. The proposed model includes the 2D-DWT signal processing method based on the “sym7” wavelet family, the wrapper approach consisting of FPA and SVM, and the CNN classifier. The efficiency of the proposed optimization algorithm is also compared with the PSO algorithm. The performance of the proposed CNN classifier for the hybrid model is compared with the performances of the SVM and KNN classification algorithms, and its effect on the performance of the plant leaf disease classification model is examined.

### 3.1. Dataset

In this study, a data set consisting of leaf images of apple, grape, and tomato plants with a size of 256×256 pixels was used. The image dataset consisting of apple, grape, and tomato plant diseases used in the study was randomly divided into two independent datasets, 80% and 20%, respectively, for the training and validation phases.

**Figure 3:**
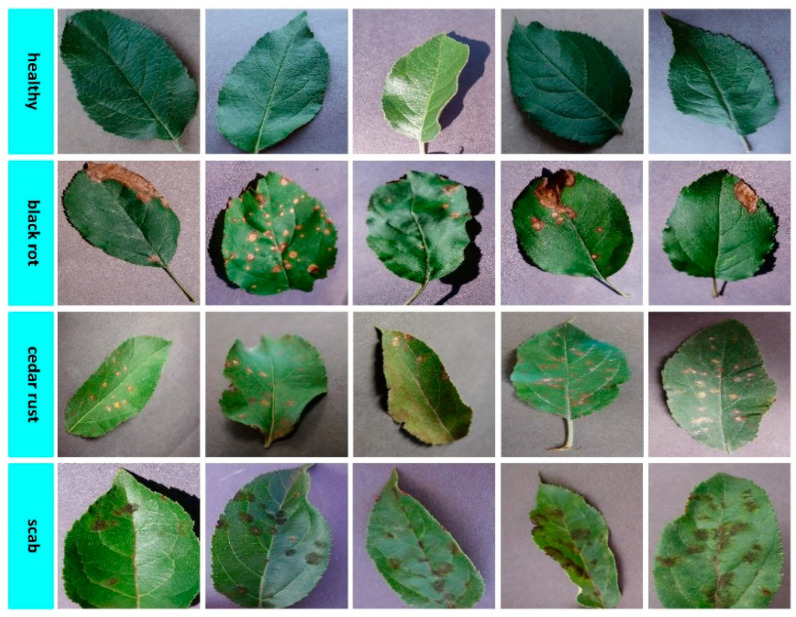
Leaf image examples belonging to healthy and diseased classes of apple plants.

**Figure 4:**
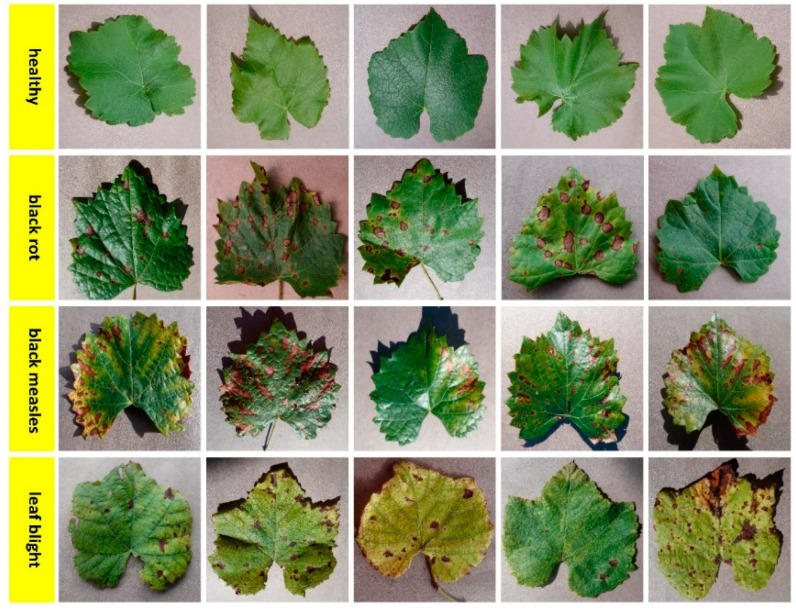
Leaf image examples belonging to healthy and diseased classes of grape plants.

**Figure 5:**
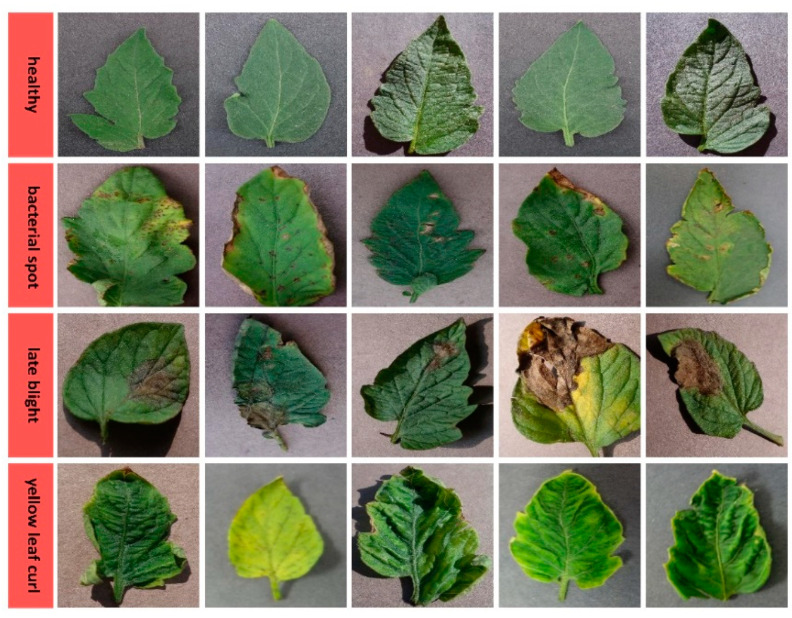
Leaf image examples belonging to healthy and diseased classes of tomato plants.

The dataset consisting of apple plant leaf images consists of healthy, black rot, cedar rust, and scab disease classes seen in Figure 3. In the training phase of the models in the study, a total of 1100 apple plant leaf images, 275 images for each class, were used, and in the validation phase, a total of 220 apple plant leaf images, 55 images for each class, were used. The dataset consisting of grape plant leaf images consists of healthy, black rot, black measles, and leaf blight disease classes shown in Figure 4. In the training phase of the models in the study, a total of 1680 grape plant leaf images, 420 images for each class, were used, and in the validation phase, a total of 336 grape plant leaf images, 84 images for each class, were used. The dataset consisting of tomato plant leaf images consists of healthy, bacterial spot, late blight, and yellow leaf curl disease classes presented in Figure 5. In the training phase of the models in the study, a total of 1700 tomato plant leaf images, 425 images for each class, were used, and in the validation phase, a total of 340 tomato plant leaf images, 85 images from each class, were used.

There are 12 classes in total in the study, and the number of plant leaf images belonging to these 12 classes is 5376, of which 4480 are training data and 896 are validation data. Apple, grape, and tomato plant leaf disease images used in the training and validation phases of the model have been taken from the open-source Plant Village dataset [5].

The testing phase of the models used in the study was carried out on the images obtained with the help of the camera on the UAV. In the study, the GoPro Hero4 camera, which can shoot 30 frames per second, was used. The images obtained with the UAV have a resolution of 1920×1080. These images were resized on the embedded system and presented to the model with 256×256 resolution. The stabilization of the camera was provided with the help of the gimbal system. In order to cope with environmental conditions, such as blurring, threshold values were used for images taken with the UAV. These threshold values decide whether the image received by the UAV will be processed or not.

The testing phase of the models was done with data as much as with the number of images used in the validation phase of the models. Real-time tests of the aforementioned models were carried out in various provinces of Turkey in August 2022. Apple leaf disease tests were carried out in apple orchards in Eskişehir, grape leaf disease tests were carried out in vineyards in Manisa, and tomato leaf disease tests were carried out in tomato fields in Antalya province. During the time intervals when the tests were carried out, the average temperature in Eskişehir was 27 ℃, the average temperature in Manisa was 30 ℃, and the average temperature in Antalya was 32 ℃. Average humidity values were measured at 62%, 73%, and 78% for Eskişehir, Manisa, and Antalya, respectively. Considering the seasonality of some diseases, the tests in the study were carried out in natural agricultural areas instead of greenhouses in various provinces of Turkey.

### 3.2. Applying 2D-DWT with Wavelet Families

The features of plant leaf diseases were extracted by 2D-DWT using the biorthogonal, Coiflet, Daubechies, Fejer–Korovkin, and symlet wavelet families for filter lengths shown in Table 2. A two-level decomposition was applied to the original image matrix of 256×256 pixels. As a result of this decomposition, vertical, horizontal, diagonal, and approximation image matrices were obtained for each wavelet family. In the first level of decomposition, four image matrices with 130×130 pixel size and four image matrices with 67×67 pixel size in the second level of decomposition, a total of eight image matrices were obtained.

### 3.3. Extraction of Statistical and Entropy-Based Features

Plant leaf disease is diagnosed by tests such as enzyme-linked immunosorbent assay (ELISA) and polymerase chain reaction (PCR) performed in the laboratory environment, as well as visual inspections made by experienced individuals, be they a botanist or farmer. While this is the right approach, it is a costly and highly labor-intensive process as it requires the installation of laboratory equipment. However, these traditional methods based on experience and laboratory testing are not suitable for real-time detection of plant leaf disease as they are time-consuming and allow expert error under heavy workload. Since microscopic evaluation and diagnostic experiments such as ELISA and PCR do not allow real-time detection of plant leaf disease, the distinguishing features of diseases have been extracted. Extracted features define the characteristic structure of plant leaf diseases, unlike the features obtained by visual image analysis techniques.

Statistical and entropy-based features were used in the study to classify plant leaf diseases with high accuracy. A total of 96 features were extracted, and each feature was labeled as seen in Table 3.

Statistical and entropy-based features given in Table 4 were applied to the image matrices, $I=\left\{I_{V}^{1},I_{H}^{1},\;I_{D}^{1},\;I_{A}^{1},\;I_{V}^{2},\;I_{H}^{2},\;I_{D}^{2},\;I_{A}^{2}\right\}$, obtained from first and second-level decomposition for each wavelet family. Half of the extracted features consist of the mean, standard deviation, and entropy values of MRV, which is the maximum row vector of the $I_{j}^{i}\left(x,y\right)$ image, and MCV, which is the maximum column vector of the $I_{j}^{i}\left(x,y\right)$ image. Thus, the sensitivity of the proposed plant leaf disease classification model to the changes in the rows and columns of the $I_{j}^{i}\left(x,y\right)$ image matrix was increased [41].

### 3.4. Feature Selection with FPA-SVM Method

In order to keep both the classification performance high and the computational complexity to a minimum level, it is necessary to determine the subset consisting of the most suitable features out of the 96 features extracted from eight image matrices obtained by applying the 2D-DWT technique. FPA, which mimics the reproduction process of flowering plants, selects the most suitable features that keep the SVM classifier performance high among the normalized features for each wavelet family with the help of the fitness function, which is defined as:

(41) $$fitness=\mu \times \gamma_{R}\left(\varepsilon_{C}\right)+\sigma \times \left(\frac{cardinality\;of\;the\;selected\;subset}{total\;number\;of\;features}\right)$$

where $\gamma_{R}\left(\varepsilon_{C}\right)$ indicates the classification error rate of classifier. μ and σ represent the significance of classification quality and subset length, respectively. Here, μ∈[0,1] and σ=(1−μ). The fitness function considers both the number of features used in the model and model performance to keep model complexity and computational cost to minimum levels.

Due to its high performance for the selected feature subset and fast response time, the wrapper approach in the study is composed of the SVM algorithm based on statistical learning theory. The effectiveness of the proposed optimization algorithm for the wrapper approach to feature selection is emphasized by comparing it with the PSO algorithm. The parameter values of the FPA-SVM and PSO-SVM wrapper approaches used in the study are presented in Table 5. It should be noted that in the models created for each wavelet family, feature selection is performed according to the parameter values in Table 5.

**Figure 6:**
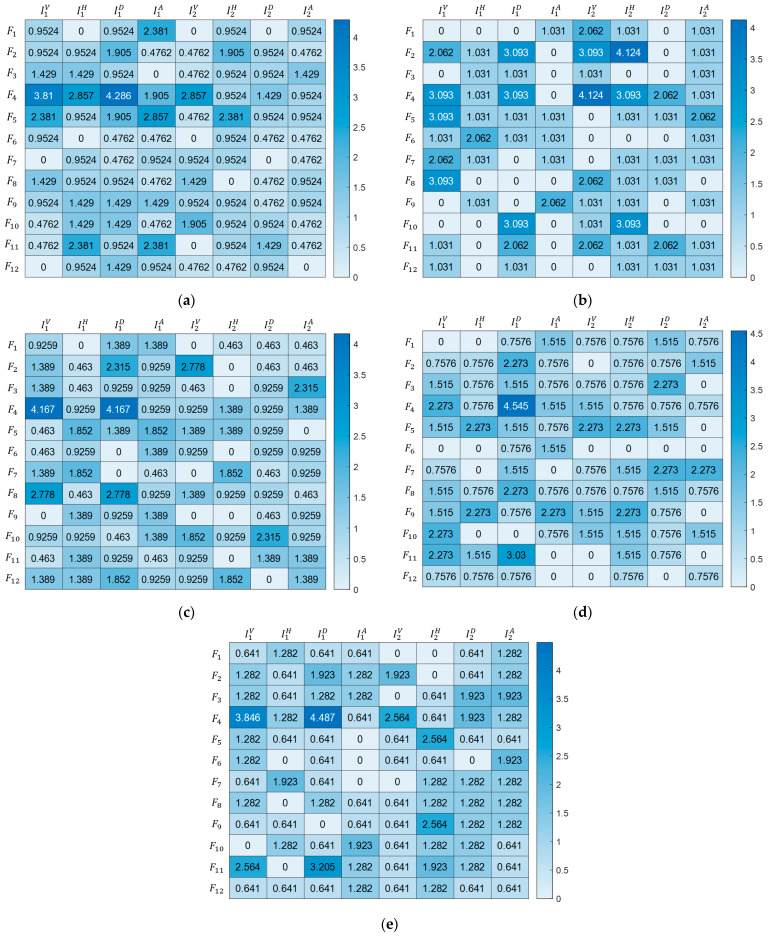
Heat map of selected features of all iterations of FPA for the (**a**) biorthogonal, (**b**) Coiflet, (**c**) Daubechies, (**d**) Fejer–Korovkin, and (**e**) symlet wavelet families.

The total selection rates for the best feature groups belonging to the biorthogonal, Coiflet, Daubechies, Fejer–Korovkin, and symlet wavelet families after 50 iterations in FPA were given as percentages in the heat map in Figure 6. It is seen that the highest number of features were extracted from $I_{H}^{1}$, $I_{D}^{1}$, and $I_{A}^{1}$ for biorthogonal; $I_{V}^{1}$, $I_{V}^{2}$, and $I_{H}^{2}$ for Coiflets; $I_{V}^{1}$, $I_{D}^{1}$, and $I_{A}^{1}$ for Daubechies; $I_{V}^{1}$, $I_{D}^{1}$, and $I_{H}^{2}$ for Fejer–Korovkin; and $I_{V}^{1}$, $I_{D}^{1}$, and $I_{H}^{2}$ for symlets in eight image matrices obtained from 2D-DWT analysis.

The $F_{4}$ feature, one of the statistical and entropy-based features, was used at least 10% in all wavelet families. In addition to this feature, $F_{5}$ for biorthogonal,$F_{2}$ for Coiflets, $F_{8}$ for Daubechies, $F_{5}$ for Fejer–Korovkin, and $F_{11}$ for symlets were selected at least 10%. It can be seen from Figure 6 that these two features specified for each wavelet family constitute at least 25% of the features selected for the plant leaf disease classification model. On the other hand, in the proposed plant leaf disease classification model, it is noted that the features $F_{6}$ and $F_{7}$ for biorthogonal; $F_{1}$, $F_{3}$, and $F_{12}$ for Coiflets; $F_{6}$ and $F_{9}$ for Daubechies; $F_{6}$ and $F_{12}$ for Fejer–Korovkin; and $F_{1}$ and $F_{6}$ for symlets were used the least.

### 3.5. Evaluation of Plant Leaf Disease Classification Models and Discussion

All models mentioned in the study of plant leaf disease images were performed on a personal computer with an Intel Core i7–10875H processor, an 8 GB NVIDIA RTX 3070 graphics card, and 16 GB of RAM. All codes for the models were compiled by MATLAB 2021b. The models created in the study were tested on 896 pieces of data, including 12 plant leaf disease classes, taken with the camera on the UAV. All models created were run 50 times, and the performance of the models was computed as the mean and standard deviation. In the study, the effects of both optimization and classifier algorithms on model performance were examined. The performances of the CNN, KNN, and SVM classification models created with the features selected by both the FPA-SVM and PSO-SVM wrapper approaches were measured in terms of accuracy metrics, and the results are presented in Table 6 and Table 7, respectively.

Twenty-three feature subsets were selected from the features generated by the 2D-DWT method based on the “***sym7***” wavelet family by the FPA-SVM wrapper approach. The performances of CNN, SVM, and KNN classifiers created with 23 selected features have been measured at 99.55%, 97.54%, and 93.97%, respectively. The plant leaf disease classification model, which includes the 2D-DWT method based on the “***sym7***” wavelet family, the wrapper approach consisting of FPA and SVM, and the CNN classifier, has been proposed as the model with the best classification performance in the study. For the wrapper approach consisting of FPA and SVM, the best performance among the models created with the SVM and KNN classifier algorithms has been obtained with the features generated by the “***sym7***” wavelet family. The performance of the CNN classifier has been measured over 95% for wavelet families excluding “*coif2*” and “*db2*”. The performance of the SVM classifier has been measured at over 90% for wavelet families excluding “*bior1.1*” and “*db1*”. The performance of the KNN classifier has been measured at over 85% for wavelet families excluding “*bior1.1*”, “*bior1.3*”, “*bior1.5*”, “*bior2.8*”, “*db1*”, “*db2*”, “*fk4*”, and “*sym4*”. It is seen that the performance of the CNN classifier is superior when compared to the SVM and KNN classifiers.

Twenty-two feature subsets were selected from the features generated by the 2D-DWT method based on the “***db6***” wavelet family by the PSO-SVM wrapper approach. The performances of CNN, SVM, and KNN classifiers created with 22 selected features have been measured at 94.87%, 90.15%, and 87.00%, respectively. For the wrapper approach consisting of PSO and SVM, the best performance among the models created with the SVM and KNN classifier algorithms has been obtained with the features generated by the “***db6***” wavelet family. The performance of the CNN classifier has been measured over 90% for wavelet families excluding “*bior2.6*”. The performance of the SVM classifier has been measured over 85% for wavelet families, excluding “*bior1.1*”, “*bior1.3*”, “*bior1.5*”, “*bior2.6*”, “*coif5*”, “*db1*”, “*db8*”, “*db9*”, “*fk4*”, “*fk6*”, “*fk14*”, “*sym2*”, and “*sym7*”. The performance of the KNN classifier has been measured over 80% for wavelet families, excluding “*bior1.3*”, “*bior1.5*”, “*bior2.6*”, “*bior3.3*”, “*coif1*”, “*coif5*”, “*db3*”, “*db8*”, “*db9*”, “*db10*”, “*fk4*”, “*fk6*”, “*fk8*”, “*fk14*”, “*sym2*”, and “*sym7*”. It is seen that the performance of the CNN classifier is superior when compared to the SVM and KNN classifiers.

Considering the model structure and input size of CNN, it can be said that the model complexity of the proposed classification model is low since it has high performance with fewer features selected by the FPA-SVM wrapper approach. When Table 6 and Table 7 are evaluated together, considering both the classification model performance and the number of selected features, it is seen that the proposed FPA technique is successful compared to the PSO algorithm for CNN, SVM, and KNN classifiers.

**Figure 7:**
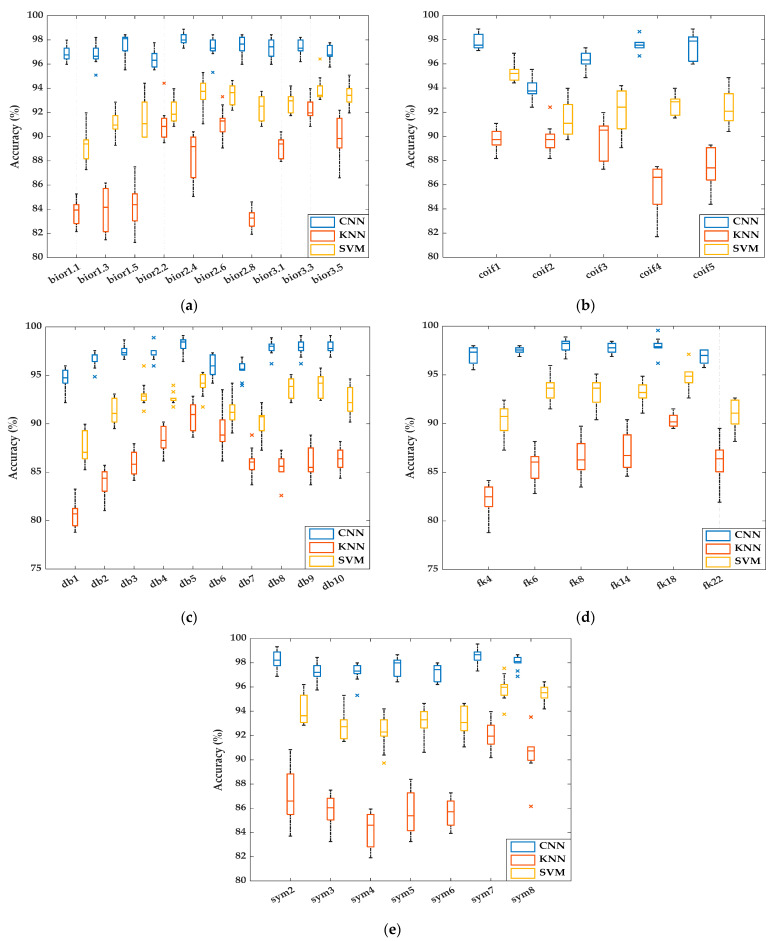
Performances of classifiers used in the study for the (**a**) biorthogonal, (**b**) Coiflet, (**c**) Daubechies, (**d**) Fejer–Korovkin, and (**e**) symlet wavelet families.

The CNN, SVM, and KNN classifier model performances, which were obtained as a result of the 50 times training-validation process for the selected features with the FPA-SVM wrapper approach proposed in the study, are compared with each other for the “*bior*”, “*coif*”, “*db*”, “*fk*”, and “*sym*” wavelet families in Figure 7.

It is seen that the performances of the models obtained with the CNN classifier are obviously higher than the performances of the models obtained with both SVM and KNN classifiers. It should also be noted that the SVM classifier performance is higher than the KNN classifier performance for the specified wavelet families. The performance of all classifiers created with the selected features with the FPA-SVM wrapper approach proposed for the “*sym7*” wavelet family has the best performance. It is noteworthy that the proposed FPA optimization technique improves CNN classifier performance by approximately 5% and SVM and KNN classifier performances by approximately 8% compared to the PSO. It is also seen that the performance of all classifiers created with the features selected with the PSO-SVM wrapper approach for the “*db6*” wavelet family has the best performance. The performances of various classifiers created with the features selected by the FPA-SVM and PSO-SVM wrapper approaches for wavelet families that provide the best classification performance are measured with the precision, recall, and F1 score performance metrics as well as the accuracy metric and are presented in Table 8.

**Figure 8:**
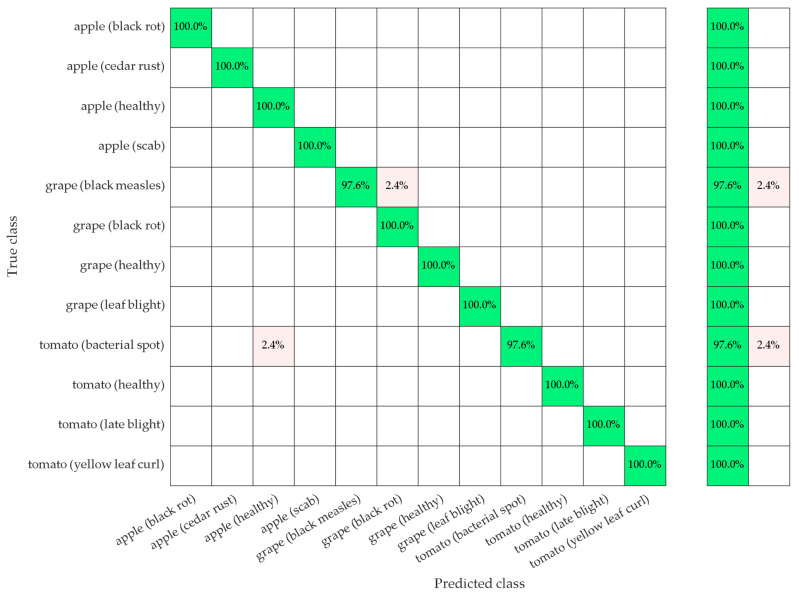
Confusion matrix for the proposed apple, grape, and tomato plant leaf disease classification model.

In the study, the best-performing model, which includes the 2D-DWT signal processing method based on the “*sym7*” wavelet family and the wrapper approach consisting of FPA-SVM and the CNN classifier, was embedded in the NVIDIA Jetson Nano artificial intelligence (AI) application development kit on the UAV, and real-time classification tests have been performed on apple, grape, and tomato plants. The results obtained as a result of the experiments performed on the test images are presented in Figure 8 in the form of a confusion matrix. It is seen that the model run in real time has a very low-level classification error for only two disease classes out of 12. The model predicts 2 out of 84 grape (black measles) test data as grape (black rot) disease classes. Similarly, the model classifies 2 out of 85 tomato (bacterial spot) test data as apple (healthy). The proposed model can classify both plant species and plant diseases in real time with high accuracy.

Moreover, a total of 227 models created, except for the model proposed in the study, were embedded on the NVIDIA Jetson Nano AI application development kit on the UAV, and the performances of all models were measured. When the performances of the models were evaluated in terms of computational time, it was seen that the computational time of the proposed model was 8.1% better than the second-best model in the study. As a result of the experimental studies, it is concluded that our robust hybrid classification model, which is created by selecting the lowest number of features with an optimization algorithm with low computational complexity, can classify plant leaf diseases with precision in real time.

## 4. Conclusions and Future Work

Detection of plant diseases at an early phase is of great importance for appropriate treatment. Automatic detection and classification of plant leaf diseases instead of visual inspection contributes to increasing agricultural product productivity. In this study, a new robust hybrid classification model based on swarm optimization-supported feature selection, including machine learning and deep learning algorithms, is proposed to classify diseases in apple, grape, and tomato plants with high accuracy in real time. The proposed model includes the 2D-DWT signal processing method based on the “*sym7*” wavelet family, the wrapper approach consisting of FPA-SVM, and the CNN classifier. In order for the model to be robust, features extracted from various wavelet families, such as biorthogonal, Coiflets, Daubechies, Fejer–Korovkin, and symlets, are used with the 2D-DWT method. The features that keep the classifier performance high for each wavelet family are selected with a wrapper approach consisting of FPA and SVM, so that the computational complexity of the model has been kept to a minimum level. The CNN classification model is created using the lowest number of features that can keep the classification performance high. The CNN classifier with a single layer of classification without a feature extraction layer is used to minimize the complexity of the model and to overcome the model hyperparameter problem. Our proposed model for plant leaf disease classification is embedded in the NVIDIA Jetson Nano AI development kit on the UAV, and its real-time performance is tested. The experimental results obtained prove that the proposed model can detect and classify the specified plant leaf diseases in real time with high accuracy. The proposed classification model with fast and low computational cost will contribute to an increase in agricultural efficiency by classifying plant leaves with high accuracy at an early phase. In future studies, algorithms that can cope with the chaoticity on 2D images will be tested on classifier model structures in order to detect and classify non-specific diseases in similar leaf images.

## Figures and Tables

**Table 1:** Scaling and mother wavelet functions of wavelets that provide the best classification performance for each wavelet type used in the study.

*Wavelet Types*	*Scaling Function* φ(x,y)=φ(x)φ(y)	*Horizontal Wavelet* $\psi^{H}\left(x,y\right)=\psi \left(x\right)\varphi \left(y\right)$	*Vertical Wavelet* $\psi^{V}\left(x,y\right)=\varphi \left(x\right)\psi \left(y\right)$	*Diagonal Wavelet* $\psi^{D}\left(x,y\right)=\psi \left(x\right)\psi \left(y\right)$
***Biorthogonal spline***, *bior2.4*	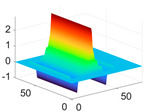	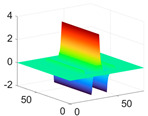	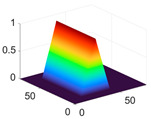	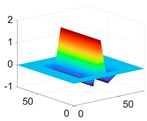
***Coiflets***, coif1	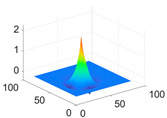	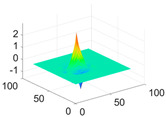	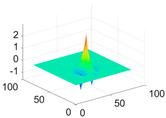	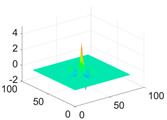
***Daubechies***, db5	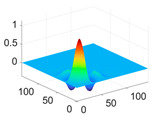	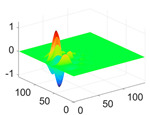	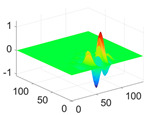	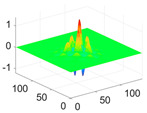
***Fejer-Korovkin***, fk18	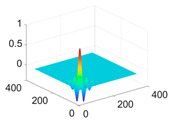	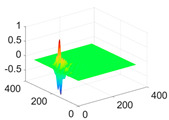	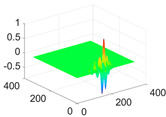	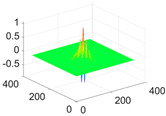
***Symlets***, sym7	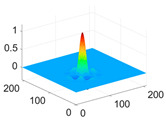	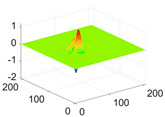	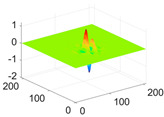	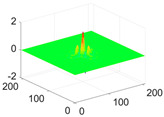

**Table 2:** Wavelet families and filter lengths used in the study.

Wavelet Family	Filter Length
Biorthogonal	(1.) 1, 3, 5, (2.) 2, 4, 6, 8, (3.) 1, 3, 5
Coiflet	1, 2, 3, 4, 5
Daubechies	1, 2, 3, 4, 5, 6, 7, 8, 9, 10
Fejer–Korovkin	4, 6, 8, 14, 18, 22
Symlet	2, 3, 4, 5, 6, 7, 8

**Table 3:** Index labels of features extracted from image matrices.

	$I_{V}^{1}$	$I_{H}^{1}$	$I_{D}^{1}$	$I_{A}^{1}$	$I_{V}^{2}$	$I_{H}^{2}$	$I_{D}^{2}$	$I_{A}^{2}$
$F_{1}$	1	13	25	37	49	61	73	85
$F_{2}$	2	14	26	38	50	62	74	86
$F_{3}$	3	15	27	39	51	63	75	87
$F_{4}$	4	16	28	40	52	64	76	88
$F_{5}$	5	17	29	41	53	65	77	89
$F_{6}$	6	18	30	42	54	66	78	90
$F_{7}$	7	19	31	43	55	67	79	91
$F_{8}$	8	20	32	44	56	68	80	92
$F_{9}$	9	21	33	45	57	69	81	93
$F_{10}$	10	22	34	46	58	70	82	94
$F_{11}$	11	23	35	47	59	71	83	95
$F_{12}$	12	24	36	48	60	72	84	96

**Table 4:** Statistical and entropy-based features and their equations.

Label	Feature Name	Feature Expression
$F_{1}$	*Arithmetic mean*	$mean=\frac{1}{m\times n}\overset{m}{\underset{x}{\sum }}\overset{n}{\underset{y}{\sum }}\left|I_{j}^{i}\left(x,y\right)\right|$
$F_{2}$	*Entropy*	$entropy=\overset{m}{\underset{x}{\sum }}\overset{n}{\underset{y}{\sum }}I_{j}^{i}\left(x,y\right)log\left|I_{j}^{i}\left(x,y\right)\right|$
$F_{3}$	*Standard deviation*	$std=\sqrt{\frac{1}{m\times n}\overset{m}{\underset{x}{\sum }}\overset{n}{\underset{y}{\sum }}{\left(\left|I_{j}^{i}\left(x,y\right)\right|-mean\right)}^{2}}$
$F_{4}$	*Skewness*	$skw=\frac{1}{m\times n}\overset{m}{\underset{x}{\sum }}\overset{n}{\underset{y}{\sum }}{\left(\frac{\left|I_{j}^{i}\left(x,y\right)\right|-mean}{std}\right)}^{3}$
$F_{5}$	*Kurtosis*	$krts=\frac{1}{m\times n}\overset{m}{\underset{x}{\sum }}\overset{n}{\underset{y}{\sum }}{\left(\frac{\left|I_{j}^{i}\left(x,y\right)\right|-mean}{std}\right)}^{4}$
$F_{6}$	*Energy*	$energy=\sqrt{\overset{m}{\underset{x}{\sum }}\overset{n}{\underset{y}{\sum }}{\left(I_{j}^{i}\left(x,y\right)\right)}^{2}}$
$F_{7}$	*MRV mean*	$MRV_{mean}=\frac{1}{m}\overset{m}{\underset{x}{\sum }}MRV\left(x\right)$
$F_{8}$	*MCV mean*	$MCV_{mean}=\frac{1}{n}\overset{n}{\underset{y}{\sum }}MCV\left(y\right)$
$F_{9}$	*Standard deviation of MRV*	$MRV_{std}=\sqrt{\frac{1}{m}\overset{m}{\underset{x}{\sum }}{\left(MRV\left(x\right)-MRV_{mean}\right)}^{2}}$
$F_{10}$	*Standard deviation of MCV*	$MCV_{std}=\sqrt{\frac{1}{n}\overset{n}{\underset{y}{\sum }}{\left(MCV\left(y\right)-MCV_{mean}\right)}^{2}}$
$F_{11}$	*MRV entropy*	$MRV_{entropy}=\overset{m}{\underset{x}{\sum }}\left|MRV\left(x\right)\right|log\left|MRV\left(x\right)\right|$
$F_{12}$	*MCV entropy*	$MCV_{entropy}=\overset{n}{\underset{y}{\sum }}\left|MCV\left(y\right)\right|log\left|MCV\left(y\right)\right|$

**Table 5:** Parameter values of wrapper feature selection approaches used in the study.

Parameters	FPA-SVM	PSO-SVM
*Number of solutions*	30	30
*Maximum number of iterations*	50	50
*Number of features*	96	96
*Threshold*	0.7	0.7
*Other parameters*	*switch probability* = 0.4 *levy component* = 1.5	*cognitive factor* = 2 *social factor* = 2 *inertia weight* = 1
*Fitness function*	*maximization of classifier performance* & *minimization of the number of selected features*	

**Table 6:** The performance of the classifiers for selected features by the FPA-SVM wrapper approach.

Wavelets		Num.	Selected Features	Accuracy (%)		
				CNN	SVM	KNN
Biorthogonal (*bior)*	1.1	24	3, 4, 5, 14, 15, 16, 23, 24, 26, 33, 36, 45, 47, 48, 55, 56, 65, 75, 80, 85, 87, 88, 89, 92	96.90 ± 0.09	89.26 ± 0.36	83.66 ± 0.04
	1.3	23	8, 17, 20, 22, 23, 25, 26, 28, 32, 37, 41, 44, 45, 52, 58, 60, 62, 63, 65, 74, 77, 84, 85	96.76 ± 0.11	91.05 ± 0.02	83.88 ± 0.07
	1.5	26	3, 4, 6, 15, 16, 21, 22, 27, 28, 29, 34, 37, 40, 43, 45, 48, 63, 64, 65, 69, 71, 76, 83, 90, 91, 94	97.68 ± 0.69	91.56 ± 0.63	84.22 ± 0.16
	2.2	17	4, 6, 28, 29, 34, 35, 41, 47, 50, 52, 55, 61, 62, 64, 65, 76, 83	96.45 ± 0.20	92.14 ± 0.27	91.00 ± 0.96
	2.4	21	1, 4, 5, 8, 23, 27, 28, 30, 34, 36, 41, 42, 47, 56, 57, 58, 65, 66, 87, 92, 93	98.08 ± 0.02	93.53 ± 0.33	88.50 ± 0.78
	2.6	19	4, 5, 9, 11, 16, 21, 23, 28, 31, 32, 33, 37, 40, 41, 52, 53, 57, 58, 75	97.34 ± 0.47	93.53 ± 0.11	91.12 ± 0.07
	2.8	21	10, 17, 19, 25, 28, 29, 33, 35, 37, 41, 47, 66, 67, 69, 70, 81, 82, 83, 84, 88, 95	97.50 ± 0.29	92.37 ± 0.07	83.21 ± 0.04
	3.1	20	1, 2, 3, 4, 5, 9, 15, 16, 19, 24, 26, 28, 40, 52, 61, 67, 70, 76, 82, 89	97.34 ± 0.13	92.90 ± 0.07	89.13 ± 0.04
	3.3	19	4, 5, 8, 16, 23, 26, 28, 29, 38, 52, 56, 62, 71, 74, 77, 78, 86, 87, 93	97.34 ± 0.13	93.95 ± 0.80	92.21 ± 0.20
	3.5	20	2, 4, 14, 16, 20, 21, 22, 28, 36, 37, 40, 41, 43, 46, 47, 51, 52, 58, 62, 72	96.90 ± 0.13	93.39 ± 0.13	90.00 ± 0.60
Coiflets (*coif*)	1	24	4, 5, 8, 11, 14, 15, 16, 19, 28, 34, 35, 49, 50, 52, 58, 61, 62, 64, 68, 70, 84, 85, 89, 91	97.77 ± 0.22	95.29 ± 0.36	89.69 ± 0.07
	2	14	2, 4, 12, 28, 34, 41, 50, 52, 59, 62, 64, 76, 83, 93	93.86 ± 0.11	91.38 ± 0.47	89.87 ± 0.42
	3	18	2, 4, 5, 6, 7, 8, 26, 29, 43, 45, 52, 56, 62, 64, 70, 76, 79, 96	96.32 ± 0.22	92.17 ± 0.54	89.89 ± 0.27
	4	23	7, 8, 17, 18, 21, 26, 28, 34, 35, 37, 42, 45, 49, 56, 57, 62, 65, 67, 80, 86, 88, 90, 95	97.57 ± 0.09	92.63 ± 0.11	85.87 ± 1.27
	5	19	4, 5, 18, 26, 27, 30, 36, 50, 51, 52, 59, 69, 70, 71, 72, 77, 83, 87, 89	97.54 ± 0.11	92.37 ± 0.27	87.30 ± 0.47
Daubechies (*db*)	1	21	5, 8, 17, 18, 19, 25, 26, 28, 35, 36, 37, 44, 45, 53, 56, 65, 72, 77, 82, 83, 95	94.60 ± 0.51	87.48 ± 0.13	80.71 ± 0.31
	2	22	2, 4, 11, 12, 16, 17, 20, 24, 27, 32, 37, 45, 52, 58, 59, 60, 64, 67, 78, 80, 88, 96	96.70 ± 0.49	91.27 ± 0.02	84.02 ± 0.65
	3	23	3, 4, 7, 15, 19, 21, 25, 28, 35, 39, 42, 50, 51, 52, 53, 67, 68, 75, 78, 80, 81, 93, 96	97.43 ± 0.22	93.04 ± 0.60	85.94 ± 0.11
	4	19	4, 12, 23, 26, 28, 29, 33, 34, 36, 37, 50, 53, 58, 60, 65, 67, 70, 87, 95	97.34 ± 0.09	92.59 ± 0.27	88.44 ± 0.27
	5	21	3, 4, 8, 14, 23, 26, 28, 32, 40, 41, 46, 48, 50, 58, 65, 70, 72, 75, 82, 83, 87	98.19 ± 0.42	94.11 ± 0.58	90.67 ± 0.04
	6	19	2, 4, 10, 21, 26, 28, 32, 38, 39, 41, 46, 47, 64, 72, 76, 82, 83, 91, 93	95.92 ± 0.16	91.38 ± 0.25	89.24 ± 0.60
	7	18	4, 6, 7, 8, 17, 19, 21, 23, 28, 32, 33, 36, 41, 58, 61, 64, 72, 85	95.63 ± 0.20	90.29 ± 0.56	86.05 ± 0.22
	8	27	1, 2, 4, 8, 12, 16, 22, 24, 25, 27, 28, 29, 32, 42, 50, 54, 56, 67, 73, 76, 82, 87, 88, 92, 94, 95, 96	97.88 ± 0.33	93.62 ± 0.02	85.60 ± 0.67
	9	24	3, 4, 7, 8, 10, 22, 28, 29, 32, 40, 41, 43, 45, 48, 50, 54, 56, 59, 74, 79, 87, 90, 91, 94	97.88 ± 0.22	93.93 ± 0.16	86.07 ± 0.20
	10	22	1, 4, 8, 17, 18, 19, 24, 26, 28, 36, 38, 42, 44, 46, 50, 68, 77, 82, 86, 87, 88, 90	97.88 ± 0.11	92.30 ± 0.11	86.41 ± 0.13
Fejer-Krovkin (*fk*)	4	24	7, 8, 9, 16, 17, 20, 21, 23, 26, 28, 35, 37, 42, 45, 57, 61, 65, 69, 75, 79, 80, 86, 88, 96	96.99 ± 0.22	90.31 ± 0.47	82.19 ± 0.71
	6	20	4, 5, 10, 11, 23, 27, 28, 30, 31, 32, 33, 37, 44, 52, 53, 55, 57, 58, 71, 75	97.52 ± 0.09	93.62 ± 0.13	85.69 ± 0.20
	8	23	3, 4, 8, 11, 21, 28, 32, 35, 38, 39, 40, 42, 45, 56, 67, 72, 73, 77, 81, 83, 91, 92, 94	97.97 ± 0.20	93.21 ± 0.47	86.58 ± 0.02
	14	21	3, 10, 11, 15, 17, 24, 26, 28, 29, 31, 32, 49, 53, 67, 68, 75, 76, 77, 80, 82, 91	97.70 ± 0.04	93.15 ± 0.18	87.17 ± 0.33
	18	23	4, 5, 9, 10, 12, 14, 17, 21, 25, 26, 27, 28, 35, 52, 62, 63, 65, 69, 70, 71, 73, 74, 79	97.99 ± 0.11	94.75 ± 0.11	90.33 ± 0.18
	22	21	2, 28, 29, 35, 36, 40, 41, 45, 46, 51, 53, 58, 64, 65, 69, 70, 79, 85, 86, 91, 94	96.81 ± 0.16	90.96 ± 0.56	86.03 ± 0.31
**Symlets (*sym*)**	2	25	2, 4, 11, 13, 17, 22, 24, 28, 29, 35, 38, 64, 65, 69, 71, 72, 75, 76, 79, 80, 81, 87, 90, 92, 93	98.28 ± 0.18	94.13 ± 0.40	87.14 ± 0.13
	3	21	2, 4, 6, 7, 8, 9, 19, 25, 28, 32, 35, 46, 52, 59, 65, 71, 75, 81, 84, 85, 89	97.21 ± 0.11	92.95 ± 0.47	85.83 ± 0.45
	4	20	3, 5, 11, 15, 19, 26, 27, 28, 35, 50, 60, 68, 69, 70, 74, 75, 76, 85, 87, 96	97.21 ± 0.56	92.28 ± 0.31	84.22 ± 0.29
	5	24	3, 4, 6, 11, 12, 14, 19, 26, 28, 31, 32, 39, 46, 48, 52, 47, 65, 66, 67, 83, 86, 88, 90, 91	97.68 ± 0.13	93.24 ± 0.60	85.65 ± 0.18
	6	20	4, 11, 27, 28, 35, 37, 38, 39, 53, 54, 56, 58, 69, 70, 71, 72, 77, 83, 86, 91	97.23 ± 0.13	93.15 ± 0.29	85.71 ± 0.11
	**7**	**23**	**1, 4, 5, 8, 16, 21, 22, 26, 28, 34, 36, 40, 44, 45, 46, 47, 50, 52, 57, 80, 82, 87, 94**	**99.55 ± 0.13**	**97.54 ± 0.25**	**93.97 ± 0.04**
	8	23	4, 13, 16, 28, 30, 35, 48, 50, 52, 63, 65, 67, 68, 69, 73, 76, 79, 82, 88, 90, 92, 93, 95	98.06 ± 0.29	95.47 ± 0.16	90.42 ± 0.58

**Table 7:** The performance of the classifiers for selected features by the PSO-SVM wrapper approach.

Wavelets		Num.	Selected Features	Accuracy (%)		
				CNN	SVM	KNN
Biorthogonal (*bior*)	1.1	19	5, 11, 19, 26, 28, 29, 32, 40, 41, 45, 56, 62, 63, 64, 70, 75, 85, 87, 96	91.71 ± 0.04	84.93 ± 1.09	80.40 ± 0.36
	1.3	21	3, 4, 5, 9, 11, 14, 15, 19, 21, 25, 27, 39, 46, 62, 63, 74, 76, 77, 84, 91, 93	92.03 ± 0.29	81.96 ± 0.02	74.28 ± 0.18
	1.5	23	3, 5, 8, 9, 10, 26, 28, 29, 31, 34, 37, 40, 41, 49, 56, 59, 62, 66, 79, 81, 87, 91, 92	92.90 ± 0.25	84.64 ± 0.54	78.17 ± 0.02
	2.2	17	1, 2, 4, 16, 26, 27, 28, 52, 53, 65, 69, 70, 78, 85, 86, 87, 96	91.11 ± 0.09	86.85 ± 0.78	85.31 ± 0.20
	2.4	18	3, 4, 5, 6, 17, 24, 26, 27, 28, 36, 40, 47, 53, 69, 76, 79, 80, 87	92.41 ± 0.02	86.42 ± 0.13	85.55 ± 0.04
	2.6	15	4, 16, 44, 47, 50, 52, 57, 60, 69, 70, 73, 76, 80, 81, 89	88.14 ± 0.18	82.34 ± 0.16	79.19 ± 0.63
	2.8	19	12, 16, 23, 28, 35, 41, 42, 43, 45, 51, 59, 66, 67, 72, 73, 76, 79, 90, 96	93.10 ± 0.45	87.32 ± 0.02	81.49 ± 0.22
	3.1	20	1, 9, 12, 14, 15, 16, 20, 24, 28, 29, 31, 32, 34, 57, 63, 77, 87, 88, 92, 94	92.96 ± 0.09	86.42 ± 0.13	82.47 ± 0.20
	3.3	21	1, 2, 6, 12, 13, 29, 35, 37, 40, 44, 52, 53, 57, 62, 64, 67, 70, 77, 83, 86, 89	92.79 ± 0.09	85.29 ± 0.33	78.23 ± 0.47
	3.5	19	2, 5, 7, 12, 14, 16, 28, 31, 38, 42, 51, 60, 66, 67, 72, 79, 83, 89, 92	92.74 ± 0.20	87.16 ± 0.65	83.21 ± 0.27
Coiflets (*coif*)	1	19	3, 4, 10, 11, 17, 35, 36, 37, 50, 56, 59, 61, 68, 73, 84, 88, 89, 92, 95	92.23 ± 0.09	85.29 ± 0.22	79.71 ± 0.22
	2	22	4, 13, 14, 15, 24, 28, 33, 35, 36, 39, 50, 52, 53, 58, 66, 68, 69, 76, 87, 89, 93, 96	92.94 ± 0.27	87.92 ± 0.18	85.58 ± 0.16
	3	20	1, 4, 6, 7, 13, 19, 21, 26, 28, 29, 45, 46, 52, 63, 68, 71, 76, 79, 82, 90	93.17 ± 0.04	89.44 ± 0.20	85.64 ± 0.02
	4	17	4, 12, 17, 20, 23, 26, 36, 46, 50, 52, 53, 56, 57, 58, 72, 79, 95	93.43 ± 0.01	89.33 ± 0.09	85.71 ± 0.02
	5	17	8, 16, 18, 20, 23, 24, 28, 31, 32, 33, 39, 42, 43, 49, 72, 76, 79	91.58 ± 0.27	84.44 ± 0.16	78.83 ± 0.02
**Daubechies (*db*)**	1	16	4, 5, 11, 17, 28, 35, 37, 39, 44, 51, 52, 56, 65, 88, 91, 95	93.70 ± 0.42	84.84 ± 0.33	80.82 ± 0.13
	2	19	8, 16, 17, 20, 27, 28, 35, 36, 37, 48, 52, 57, 60, 63, 64, 65, 80, 87, 88	91.71 ± 1.07	85.71 ± 0.20	81.83 ± 0.11
	3	19	4, 7, 8, 24, 26, 28, 32, 34, 35, 36, 46, 53, 54, 58, 61, 76, 83, 87, 91	91.40 ± 0.02	85.60 ± 0.47	78.81 ± 0.56
	4	23	4, 12, 17, 22, 23, 26, 35, 40, 41, 42, 44, 50, 51, 54, 55, 60, 62, 63, 65, 67, 72, 76, 94	94.21 ± 0.11	89.55 ± 0.36	83.54 ± 0.71
	5	21	4, 8, 17, 22, 23, 24, 26, 28, 31, 40, 43, 58, 64, 65, 67, 72, 73, 80, 81, 88, 89	93.79 ± 0.20	89.75 ± 0.56	85.69 ± 0.18
	**6**	**24**	**4, 6, 16, 17, 19, 21, 22, 26, 28, 29, 32, 35, 38, 41, 43, 53, 75, 76, 79, 80, 82, 86, 90, 91**	**94.87 ± 0.04**	**90.15 ± 0.29**	**87.00 ± 0.18**
	7	19	5, 7, 10, 12, 14, 15, 21, 26, 28, 37, 40, 50, 57, 59, 65, 83, 89, 92, 94	93.28 ± 0.38	86.87 ± 0.09	81.77 ± 0.25
	8	17	4, 14, 17, 18, 19, 24, 26, 28, 29, 32, 34, 50, 62, 65, 66, 73, 80	91.56 ± 0.25	84.10 ± 0.29	78.21 ± 0.16
	9	18	5, 22, 26, 28, 35, 36, 37, 39, 44, 46, 47, 56, 62, 77, 86, 88, 92, 93	92.65 ± 0.01	84.30 ± 0.02	79.04 ± 0.33
	10	22	5, 9, 11, 12, 14, 15, 16, 26, 36, 37, 40, 46, 47, 53, 55, 60, 68, 70, 80, 91, 92, 93	93.72 ± 0.07	85.87 ± 0.13	78.17 ± 0.31
Fejer-Krovkin (*fk*)	4	19	10, 12, 17, 28, 29, 32, 41, 43, 58, 72, 76, 78, 83, 85, 87, 88, 93, 94, 96	91.89 ± 0.47	82.63 ± 0.36	79.01 ± 0.13
	6	20	4, 7, 17, 22, 25, 32, 34, 35, 37, 39, 44, 47, 48, 53, 54, 61, 64, 80, 86, 90	90.60 ± 0.38	82.50 ± 0.11	77.09 ± 0.27
	8	21	2, 6, 8, 16, 28, 36, 39, 40, 42, 48, 51, 59, 60, 64, 68, 69, 70, 71, 72, 91, 93	92.23 ± 0.25	85.93 ± 0.42	79.24 ± 0.02
	14	16	3, 15, 19, 26, 28, 31, 32, 48, 52, 55, 57, 58, 62, 72, 77, 85	90.93 ± 0.07	84.24 ± 0.38	79.44 ± 0.16
	18	20	5, 8, 15, 16, 24, 26, 31, 34, 37, 49, 52, 61, 62, 76, 79, 80, 85, 88, 92, 94	92.88 ± 0.11	86.47 ± 0.29	80.49 ± 1.34
	22	21	4, 7, 8, 9, 20, 21, 22, 25, 26, 34, 45, 51, 52, 62, 64, 69, 75, 76, 81, 84, 95	92.74 ± 0.36	87.29 ± 0.45	81.98 ± 0.27
Symlets (*sym*)	2	20	4, 5, 9, 11, 21, 23, 26, 28, 29, 32, 35, 41, 46, 52, 67, 69, 73, 79, 80, 88	91.89 ± 0.02	83.86 ± 0.02	78.41 ± 0.63
	3	19	2, 4, 5, 12, 15, 21, 28, 37, 43, 44, 45, 51, 57, 62, 65, 73, 76, 94, 95	92.61 ± 0.38	87.18 ± 0.33	82.74 ± 0.13
	4	18	3, 17, 19, 21, 26, 28, 40, 52, 54, 60, 65, 72, 74, 79, 85, 90, 92, 95	91.56 ± 0.13	85.89 ± 0.16	80.75 ± 0.49
	5	23	10, 16, 17, 18, 21, 22, 26, 28, 29, 31, 39, 56, 64, 72, 75, 81, 82, 85, 91, 92, 93, 95, 96	93.41 ± 0.09	87.96 ± 0.33	80.96 ± 0.25
	6	16	4, 16, 17, 21, 27, 28, 37, 45, 60, 61, 66, 67, 68, 77, 80, 92	91.36 ± 0.16	86.96 ± 0.11	83.28 ± 0.22
	7	18	5, 9, 16, 19, 20, 28, 41, 47, 50, 52, 64, 67, 70, 78, 80, 78, 90, 95	90.33 ± 0.02	83.59 ± 0.02	79.50 ± 0.25
	8	15	4, 23, 28, 29, 32, 39, 46, 51, 52, 60, 67, 70, 76, 78, 88	90.13 ± 0.18	86.65 ± 0.25	84.04 ± 1.09

**Table 8:** Comparison of the performance of various classifiers created with the selected features by the proposed FPA-SVM and PSO-SVM wrapper approaches for wavelet families providing the best classification performance.

Feature Selection Method	Wavelet	Classifier	Accuracy (%)	Precision (%)	Recall (%)	F1 score (%)
PSO-SVM	db6	CNN	94.87	95.06	94.61	94.77
		SVM	90.15	90.21	89.62	89.73
		KNN	87.00	87.64	85.86	86.26
**FPA-SVM** (proposed)	**sym7**	**CNN**	**99.55**	**99.52**	**99.60**	**99.56**
		SVM	97.54	97.58	96.44	96.92
		KNN	93.97	94.25	93.82	93.84

## Data Availability

The data presented in this study are available upon request from the corresponding author.

## References

[1] Savary S., Ficke A., Aubertot J., Hollier C. (2012). Crop losses due to diseases and their implications for global food production losses and food security. Food Secur..

[2] Ons L., Bylemans D., Thevissen K., Cammue B.P. (2020). Combining biocontrol agents with chemical fungicides for integrated plant fungal disease control. Microorganisms.

[3] Tewari V.K., Pareek C.M., Lal G., Dhruw L.K., Singh N. (2020). Image processing based real-time variable-rate chemical spraying system for disease control in paddy crop. Artif. Intell. Agric..

[4] Chadha S., Sharma M., Sayyed A. (2021). Advances in sensing plant diseases by imaging and machine learning methods for precision crop protection. Microbial Management of Plant Stresses.

[5] Mohanty S.P., Hughes D.P., Salathé M. (2016). Using deep learning for image-based plant disease detection. Front. Plant Sci..

[6] Zhang N., Yang G., Pan Y., Yang X., Chen L., Zhao C. (2020). A review of advanced technologies and development for hyperspectral-based plant disease detection in the past three decades. Remote Sens..

[7] Barbedo J.G.A., Koenigkan L.V., Santos T.T. (2016). Identifying multiple plant diseases using digital image processing. Biosyst. Eng..

[8] Barbedo J.G.A. (2018). Impact of dataset size and variety on the effectiveness of deep learning and transfer learning for plant disease classification. Comput. Electron. Agric..

[9] Almadhor A., Rauf H.T., Lali M.I.U., Damaševičius R., Alouffi B., Alharbi A. (2021). AI-driven framework for recognition of guava plant diseases through machine learning from DSLR camera sensor based high resolution imagery. Sensors.

[10] Ali H., Lali M.I., Nawaz M.Z., Sharif M., Saleem B.A. (2017). Symptom based automated detection of citrus diseases using color histogram and textural descriptors. Comput. Electron. Agric..

[11] Wen D.M., Chen M.X., Zhao L., Ji T., Li M., Yang X.T. (2019). Use of thermal imaging and Fourier transform infrared spectroscopy for the pre-symptomatic detection of cucumber downy mildew. Eur. J. Plant Pathol..

[12] Singh V., Misra A.K. (2017). Detection of plant leaf diseases using image segmentation and soft computing techniques. Inf. Process. Agric..

[13] Saeed F., Khan M.A., Sharif M., Mittal M., Goyal L.M., Roy S. (2021). Deep neural network features fusion and selection based on PLS regression with an application for crops diseases classification. Appl. Soft Comput..

[14] Ozguven M.M., Altas Z. (2022). A new approach to detect mildew disease on cucumber (*Pseudoperonospora cubensis*) leaves with image processing. J. Plant Pathol..

[15] Tan L., Lu J., Jiang H. (2021). Tomato leaf diseases classification based on leaf images: A comparison between classical machine learning and deep learning methods. AgriEngineering.

[16] Kamal K.C., Yin Z., Wu M., Wu Z. (2019). Depthwise separable convolution architectures for plant disease classification. Comput. Electron. Agric..

[17] Atila Ü., Uçar M., Akyol K., Uçar E. (2021). Plant leaf disease classification using EfficientNet deep learning model. Ecol. Inform..

[18] Li Y., Nie J., Chao X. (2020). Do we really need deep CNN for plant diseases identification. Comput. Electron. Agric..

[19] Thangaraj R., Anandamurugan S., Kaliappan V.K. (2021). Automated tomato leaf disease classification using transfer learning-based deep convolution neural network. J. Plant Dis. Prot..

[20] Too E.C., Yujian L., Njuki S., Yingchun L. (2019). A comparative study of fine-tuning deep learning models for plant disease identification. Comput. Electron. Agric..

[21] Darwish A., Ezzat D., Hassanien A.E. (2020). An optimized model based on convolutional neural networks and orthogonal learning particle swarm optimization algorithm for plant diseases diagnosis. Swarm Evol. Comput..

[22] Zhong J., Huang Y. (2010). Time-frequency representation based on an adaptive short-time Fourier transform. IEEE Trans. Signal Process..

[23] Li L., Cai H., Han H., Jiang Q., Ji H. (2020). Adaptive short-time Fourier transform and synchrosqueezing transform for non-stationary signal separation. Signal Process..

[24] Kim C.H., Aggarwal R. (2000). Wavelet transforms in power systems. Part 1: General introduction to the wavelet transforms. Power Eng. J..

[25] Grossmann A., Morlet J. (1984). Decomposition of Hardy functions into square integrable wavelets of constant shape. SIAM J. Math. Anal..

[26] Mallat S.G. (1989). A theory for multiresolution signal decomposition: The wavelet representation. IEEE Trans. Pattern Anal. Mach. Intell..

[27] Keinert F. (1994). Biorthogonal wavelets for fast matrix computations. Appl. Comput. Harmon. Anal..

[28] Monzón L., Beylkin G., Hereman W. (1999). Compactly supported wavelets based on almost interpolating and nearly linear phase filters (coiflets). Appl. Comput. Harmon. Anal..

[29] Daubechies I. (1992). Ten Lectures on Wavelets.

[30] Cohen A., Sun Q. (1993). An arithmetic characterization of the conjugate quadrature filters associated to orthonormal wavelet bases. SIAM J. Math. Anal..

[31] Daubechies I. (1988). Orthonormal bases of compactly supported wavelets. Commun. Pure Appl. Math..

[32] Daubechies I. (1990). The wavelet transform, time-frequency localization and signal analysis. IEEE Trans. Inf. Theory.

[33] Nielsen M. (2001). On the construction and frequency localization of finite orthogonal quadrature filters. J. Approx. Theory.

[34] Mallat S. (1999). A Wavelet Tour of Signal Processing.

[35] Gonzalez R.C., Woods R.E. (2002). Digital Image Processing.

[36] Yang X.S. (2012). Flower Pollination Algorithm for Global Optimization. International Conference on Unconventional Computing and Natural Computation.

[37] Abdel-Basset M., El-Shahat D., El-Henawy I., Sangaiah A.K. (2018). A modified flower pollination algorithm for the multidimensional knapsack problem: Human-centric decision making. Soft Comput..

[38] Abdel-Basset M., Shawky L.A. (2019). Flower pollination algorithm: A comprehensive review. Artif. Intell. Rev..

[39] Gu J., Wang Z., Kuen J., Ma L., Shahroudy A., Shuai B., Liu T., Wang X., Wang G., Cai J. (2018). Recent advances in convolutional neural networks. Pattern Recognit..

[40] Ma C., Zhang H.H., Wang X. (2014). Machine learning for big data analytics in plants. Trends Plant Sci..

[41] Karasu S., Saraç Z. (2022). The effects on classifier performance of 2D discrete wavelet transform analysis and whale optimization algorithm for recognition of power quality disturbances. Cogn. Syst. Res..

